# Improving the photoelectrochemical water splitting performance of CuO photocathodes using a protective CuBi_2_O_4_ layer

**DOI:** 10.1038/s41598-023-32804-0

**Published:** 2023-04-08

**Authors:** Nguyen Hoang Lam, Nguyen Tam Nguyen Truong, Nam Le, Kwang-Soon Ahn, Younjung Jo, Chang-Duk Kim, Jae Hak Jung

**Affiliations:** 1 School of Chemical Engineering, Yeungnam University, 280 Daehak-ro, Gyeongsan, 38541 Republic of Korea; 2 Department of Physics, Kyungpook National University, Daegu, 41566 Republic of Korea

**Keywords:** Energy, Materials for energy and catalysis

## Abstract

A heterojunction photocathode of CuO and CuBi_2_O_4_ grown on an FTO substrate (FTO/CuO/CuBi_2_O_4_) was synthesized using hydrothermal method followed by spin coating and annealing to overcome the bottlenecks encountered by CuO in photoelectrochemical (PEC) water splitting application. The synthesis methods, morphological, structural properties, and composition of each sample under each synthesis condition are discussed in detail. The photocathode with 15 coating layers annealed at 450 °C exhibited the best PEC performance. Moreover, its current density reached 1.23 mA/cm^2^ under an applied voltage of − 0.6 V versus Ag/AgCl in a neutral electrolyte. Additionally, it exhibited higher stability than the bare CuO thin film. The bonding of CuBi_2_O_4_ on CuO resulted in close contact between the two semiconductors, helping the semiconductors support each other to increase the PEC efficiency of the photocathode. CuO acted as the electron-generating layer, and the CuBi_2_O_4_ layer helped minimize photocorrosion as well as transport the carriers to the electrode/electrolyte interface to accomplish the hydrogen evolution reaction.

## Introduction

PEC water splitting which was first introduced by Fujishima and Honda^1^ is a potential technique for hydrogen production for renewable energy applications to help gradually replace fossil fuels and thereby reduce greenhouse gas emissions. Generally, a PEC system converts incident photons from sunlight into electrical energy using specialized materials (semiconductors) immersed in an electrolyte containing redox pairs. Specifically, when the energy of incident photons is equal to or greater than the bandgap energy of a semiconductor, electron–hole (e^−^–h^+^) pairs are generated. Subsequently, electrons move to the photocathode/electrolyte interface to reduce H^+^ into hydrogen gas. Simultaneously, holes transferred to the surface of the photoanode to oxidize water molecules into oxygen gas. The overall reactions related to the water splitting mechanism are presented in Eq. (1)^2^. Furthermore, the overall reaction in PEC systems is divided into two half-cell reactions: hydrogen evolution reaction (HER, reduction at the cathode to produce hydrogen gas (Eq. 2)), and oxygen evolution reaction (OER, oxidation at the anode to produce oxygen gas (Eq. 3))^2^.

1 $$2{\text{H}}_{2} {\text{O}} + {\text{h}}\vartheta { } \to { }2{\text{H}}_{2} + {\text{O}}_{2} { }\quad \Delta {\text{G}}^{0} = 4.92\;{\text{ eV }}\left( {113\;{\text{ kcal }}\;{\text{mol}}^{ - 1} } \right)$$

2 $$2{\text{H}}^{ + } + { }4{\text{e}}^{ - } \to 2{\text{H}}_{2} {\text{ E}}_{{{\text{red}}}}^{{\text{o}}} = 0{\text{ V}}$$

3 $$2{\text{H}}_{2} {\text{O }} \to { }4{\text{H}}^{ + } + 4{\text{e}}^{ - } + {\text{ O}}_{2} {\text{ E}}_{{{\text{red}}}}^{{\text{o}}} = 1.23{\text{ V }}$$

In PEC cells, semiconductors play an important role in oxidation and reduction. Semiconductors can capture light and provide energy for chemical reactions. Additional voltage is required to supply sufficient voltage to PEC cells to drive reactions at the expected current densities^3^. Currently, numerous semiconductors have been used in PEC water splitting system such as BiVO_4_^4–6^, Fe_2_O_3_^7^, TiO_2_^8^, Si^9^, Cu_2_O^10^, CaFe_2_O_4_^11^, CuRhO_2_^12^, etc. Each material has its own properties for application to a photoanode or photocathode. Among these semiconductors, CuO has several advantages to being a photocathode, such as a narrow bandgap (1.2–2.0 eV), nontoxicity, abundant on Earth, capability to absorb light in the visible-light^13^, and a high theoretical photocurrent density (35 mA/cm^2^)^14^. However, CuO has the following drawbacks that scientists need to modify and improve: (1) The tendency of CuO to accumulate generated electrons on the electrode surface can cause self-reduction of CuO into Cu_2_O or Cu; (2) Giving their tendency for self-reduction, CuO photocathodes have low stability, which results in shortened electrode lifetimes^15^. The problems associated with CuO involve the improvement of photostability and the enhancement of lifetime. The latter is crucial for fabricating photocathodes for PEC water splitting. CuBi_2_O_4_ is a p-type metal oxide with lower theoretical photocurrent density than CuO (only 19 mA/cm^2^)^16^. Although bare CuBi_2_O_4_ has a considerably low current density of 0.3–0.5 mA/cm^2^^17,18^, it has several advantages: (1) it has a narrow bandgap (1.5–1.8 eV) that helps to absorb light in the visible range^19^; (2) it is formed from Earth-abundant elements (Cu, Bi, and O) and is therefore a low-cost material with low toxicity^20^; (3) its conduction band position is suitable for the reducing small molecules, such as CO_2_, H_2_O, and O_2_^16,21,22^; and (4) most importantly, it is more stable than other binary copper compounds (Cu_2_O or CuO) and can thus act as a protective layer in photocathodes to resist photo-corrosion^22^. Several methods have been proposed to assist the separation and transport of e^−^–h^+^ pairs in semiconductors. They include heterojunction formation, structural defect generation, and doping^23^. In particular, the heterojunction structure fulfills the requirements for ideal PEC photocathodes, such as high redox capacity, efficient charge separation, and wide light absorption range^23^. Until now, heterojunctions of CuO and CuBi_2_O_4_ have been widely studied. For example, Pulipaka et al.^24^ synthesized CuO/CuBi_2_O_4_ films via annealing at high temperatures between two cycles of electrodeposition. The obtained films generated a high current density (− 0.9 mA/ cm^2^ at 0.1 V vs. RHE). The H_2_O_2_ yield of a Gd^3+^ doped CuBi_2_O_4_/CuO heterojunction film considerably increased and was six times higher than that of an un-doped film^25^. Additionally, Zhang et al.^26^ constructed a CuO/CuBi_2_O_4_ bilayer decorated with a NiO_x_ electrocatalyst via pulse chronoamperometric deposition followed by the hydrothermal method. This photocathode exhibited a photocurrent density of 2.17 mA/ cm^2^ at 0.2 V versus RHE in a neutral electrolyte, and retained 82.5% of its photostability during 300 min of continuous illumination. In this study, we used a simple method (hydrothermal method followed by spin coating and annealing) to fabricate FTO/CuO/CuBi_2_O_4_ heterojunctions. We found that changes in thickness and crystallinity according to coating layer number and annealing temperature resulted in structural changes that improved the PEC properties of semiconductors.

## Results–discussion

**Figure 1:**
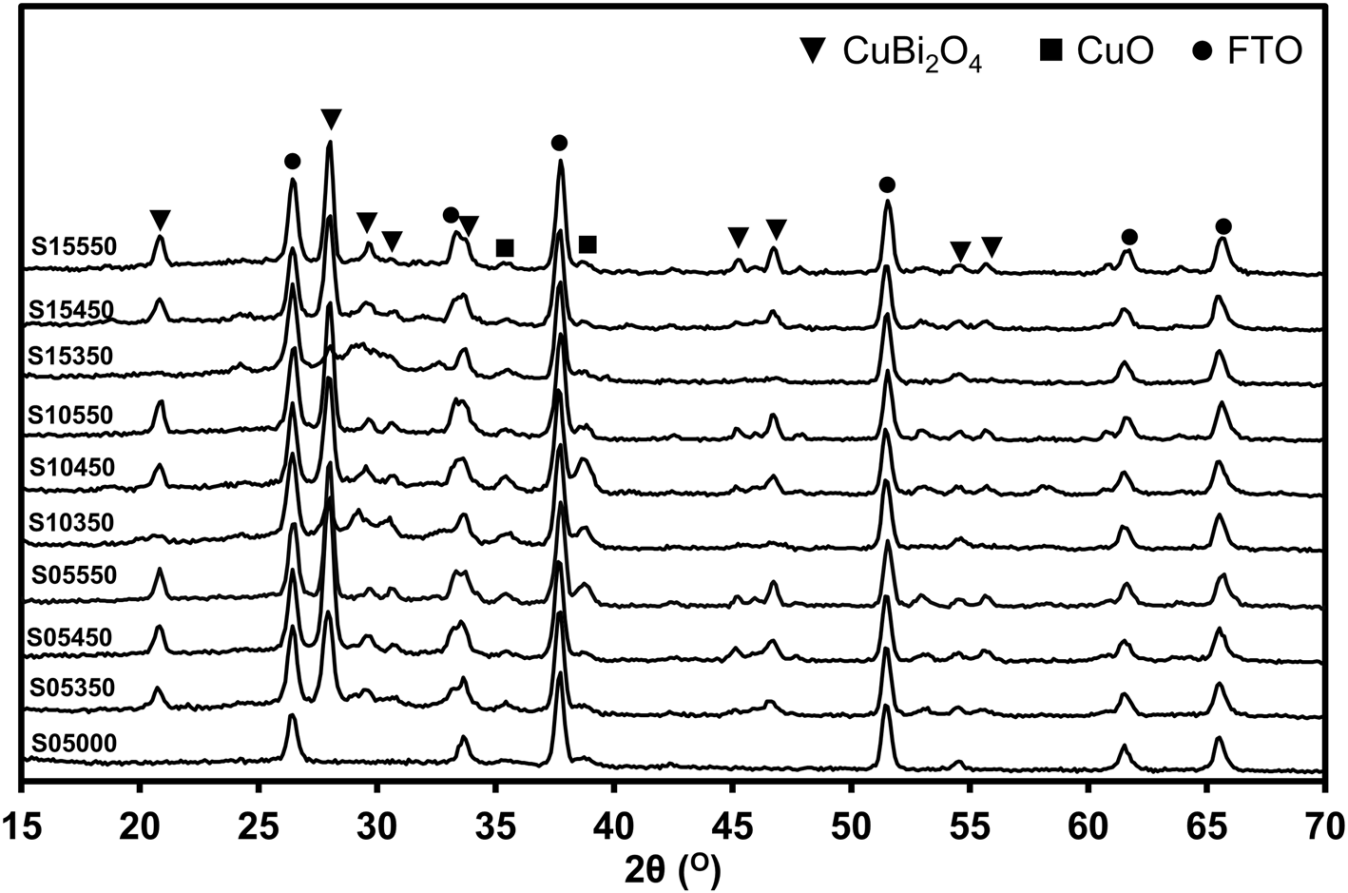
X-ray Difraction (XRD) spectra of FTO/CuO/CuBi_2_O_4_ fabricated with different numbers of coating layer at different annealing temperatures. Triangular dots represent the peaks of CuBi_2_O_4_ crystals based on JCPDS No.42.0334, and square dots represent the peaks of CuO crystals based on ICDD No#00-041-0254.

Fig. S2 in the Supplementary Information (SI) shows the X-ray Difraction (XRD) peaks of CuO, the sample with five coating layers (5CL), and the sample with five coating layers annealed at 450 °C (5CL450) grown on the surface of the FTO substrate. The spectrum of the 5CL450 sample exhibited strong peaks at approximately 20.8°, 27.9°, 29.3°, 30.6°, 46.1°, and 55.1° corresponding to the planes (2 0 0), (2 1 1), (2 2 0), (0 0 2), (4 1 1), and (3 3 2), respectively (ICDD No. 00-42-0334) indicating that CuBi_2_O_4_ layer was successfully synthesized^17,27^. Moreover, the peaks of CuO were also found in the XRD spectrum at 35.64 °, 38.90 °, and 53.62 ° corresponding to the planes (− 1 1 1), (1 1 1), (2 0 2), respectively (ICDD No. 00-041-0254)^28^. Comparing the XRD peaks of 5CL with 5CL450 revealed that the XRD diffraction peaks of CuBi_2_O_4_ appeared only after annealing. This finding demonstrated that heat treatment is an important step in crystallizing the coating into CuBi_2_O_4_. The XRD spectra of FTO/CuO/CuBi_2_O_4_ synthesized at different temperatures (350 °C, 450 °C, and 550 °C) with different numbers of coating layers (5, 10, and 15) were acquired (Fig. 1) to evaluate the relation among crystal structure, thickness, and annealing temperature. The intensities of XRD peaks changed at different temperatures. The intensities of the XRD peaks of CuBi_2_O_4_ increased with increasing temperature. Coating thickness also affected the intensity of the XRD peaks. In particular, the intensity of the planes of (2 0 0) at 20.8 ° and (2 1 1) at 27.9 ° decreased as the number of coating layers increased from 5 to 15 under heating at 350 °C because the thicker the coating, the less complete the crystallization. Therefore, the annealing temperature of 350 °C was considered to be considerably low for complete crystallization of the coatings into CuBi_2_O_4_. No considerable differences were found between the XRD spectra of the cathode annealed at 450 °C and that annealed at 550 °C regardless of the number of coating layers. Using the Scherrer’s equation (Eq. 5) to calculate the estimated crystalline size of all samples revealed that crystallite size increased with increasing temperature (Table 1). This result was in line with the results of previous studies^24,28,29^. Furthermore, the crystallization parameters revealed the structure of the FTO/CuO/CuBi_2_O_4_ film. Dislocations (Eq. 6) are anomalies in crystal structures. Similar to micro-strain (Eq. 7), dislocation in the sample tended to decrease at high temperatures. These two parameters changed inversely with crystal size, because the appearance of grain boundaries decreased owing to the increase in crystal size with increasing temperature^28,30^.

**Table 1:** Crystallographical parameters of samples.

Sample	Coating layers	Annealing temp (°C)	Crystallite size [A]	Micro-stain (%)	Dislocation density (δ)	Thickness (nm)	Band gap (eV)	Flat band (V)	Atomic percentage (%)		
									O	Cu	Bi
S05000	5	–	–	–	–	1390	–	–	53.38	37.23	9.39
S05350	5	350	199.07	0.108	3.04E−05	794	2.71	1.50	57.37	27.45	15.18
S05450	5	450	209.24	0.108	3.31E−05	675	2.66	0.40	57.06	28.15	14.79
S05550	5	550	211.52	0.099	2.47E−05	476	2.45	0.66	59.60	27.75	12.65
S10350	10	350	188.82	0.113	3.32E−05	754	1.85	0.50	54.84	30.91	14.26
S10450	10	450	200.81	0.109	3.21E−05	675	1.78	0.23	56.95	28.77	14.28
S10550	10	550	257.52	0.082	1.70E−05	675	1.70	0.66	60.43	26.76	12.81
S15350	15	350	213.57	0.102	2.79E−05	337	2.53	0.65	63.92	18.51	17.57
S15450	15	450	232.41	0.092	2.19E−05	655	1.51	0.67	60.23	22.93	16.84
S15550	15	550	250.41	0.084	1.80E−05	575	1.55	0.68	61.70	24.39	13.91

**Figure 2:**
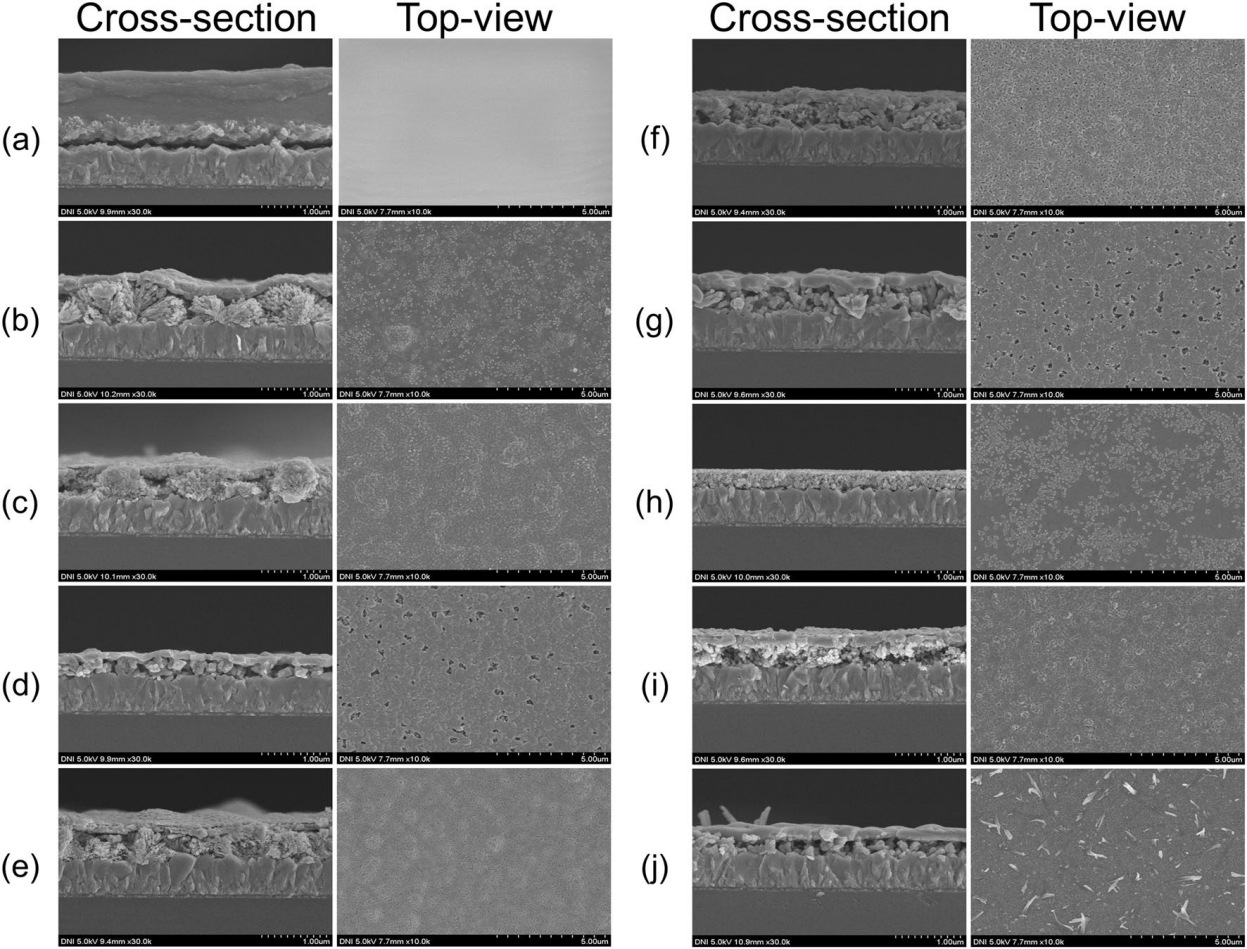
Cross-section and Planar view of the FTO/CuO/CuBi_2_O_4_ electrodes. Note: (**a**) S05000; (**b**) S05350; (**c**) S05450; (**d**) S05550; (**e**) S10350; (**f**) S10450; (**g**) S10550; (**h**) S15350; (**i**) S15450; (**j**) S15550.

Grain size and layer thickness play crucial roles in terms of the efficiency of PEC water splitting. Scanning Electron Microscopy (SEM) was performed to confirm the morphology of samples and calculate thin film thickness. Fig. 2 shows the morphology and thickness of all samples, including the FTO substrate coated with the CuO layer after the hydrothermal process. The top surfaces of the samples were covered by a CuBi_2_O_4_ layer. However, the nominal thickness of each sample differed under treatment at various temperatures with the same number of coating layers. The thickness of the sample with five coating layers on CuO was 1.39 μm (S05000). Layer thickness substantially decreased after treatment at high temperature. Specifically, the thicknesses of S05350, S05450 and S05550 were 794, 675 and 476 nm, respectively. The cross-section of S05000 showed that the two layers of CuO and CuBi_2_O_4_ had bonded together, and a continuum was found at the grain boundary of the two types of materials that will help improve the charge transfer efficiency and enable the uninterrupted transfer of generated photoelectrons to the electrode/electrolyte interface^24^. The top planar SEM image of the samples illustrated that the structure of the CuBi_2_O_4_ layer was unstable in the sample annealed at 550 °C; that voids were present in the top layer, revealing the surface of the CuO layer below, in S05550, and S10550; and that some vertical, non-uniform structures appeared on the surface of S15550. S15450 was thicker than S15550 because the thickness of S15550 was determined only to the edge of the upper CuBi_2_O_4_ layer and vertically grown crystals were excluded. In some samples annealed at lower temperatures (S05350, S10350, and S15350), the coatings were not completely converted into CuBi_2_O_4_, and discrete fractions or even discrete particles were produced. This finding was consistent with the XRD results discussed earlier. In these cases, the protective function of the CuBi_2_O_4_ layer was attenuated. The optimal annealing temperature for the formation of a flat, bonded, and homogenous surface was 450 °C, which enabled the coating layers to transform and bond with each other. SI–Fig. S3ab depicts the structure of S15450 in cross-section and planar-view, respectively. The thicknesses of the CuO and CuBi_2_O_4_ layers were 357 and 278 nm, respectively. These images also illustrate the bonding of the particles on the surface and clearly indicate the order of the layers of the material (CuO layer below and CuBi_2_O_4_ layer above). SEM – Energy-dispersive X-ray spectroscopy (EDS) was performed to confirm the chemical compositions of the samples. Bi, Cu, and O atoms were present on the surfaces of the samples (see SI–Fig. S4). The results showed that the top layer of the samples had high Cu, Bi, and O contents. Furthermore, in some samples with some voids in the top layer, the disappearance of Bi from the lost parts demonstrated that the bottom layer contained only Cu and O, indicating the presence of a layer of CuO. A precursor solution was prepared with Cu:Bi ratio of 4:1. S05000 presented correct proportion (Table 1). The formation of the heterojunction structure of CuO/CuBi_2_O_4_ on the surface of FTO substrate was confirmed on the basis of SEM images and XRD results. The heterojunction can be defined and modeled using VESTA software (version 3)^31^ (SI–Fig. S5).

**Figure 3:**
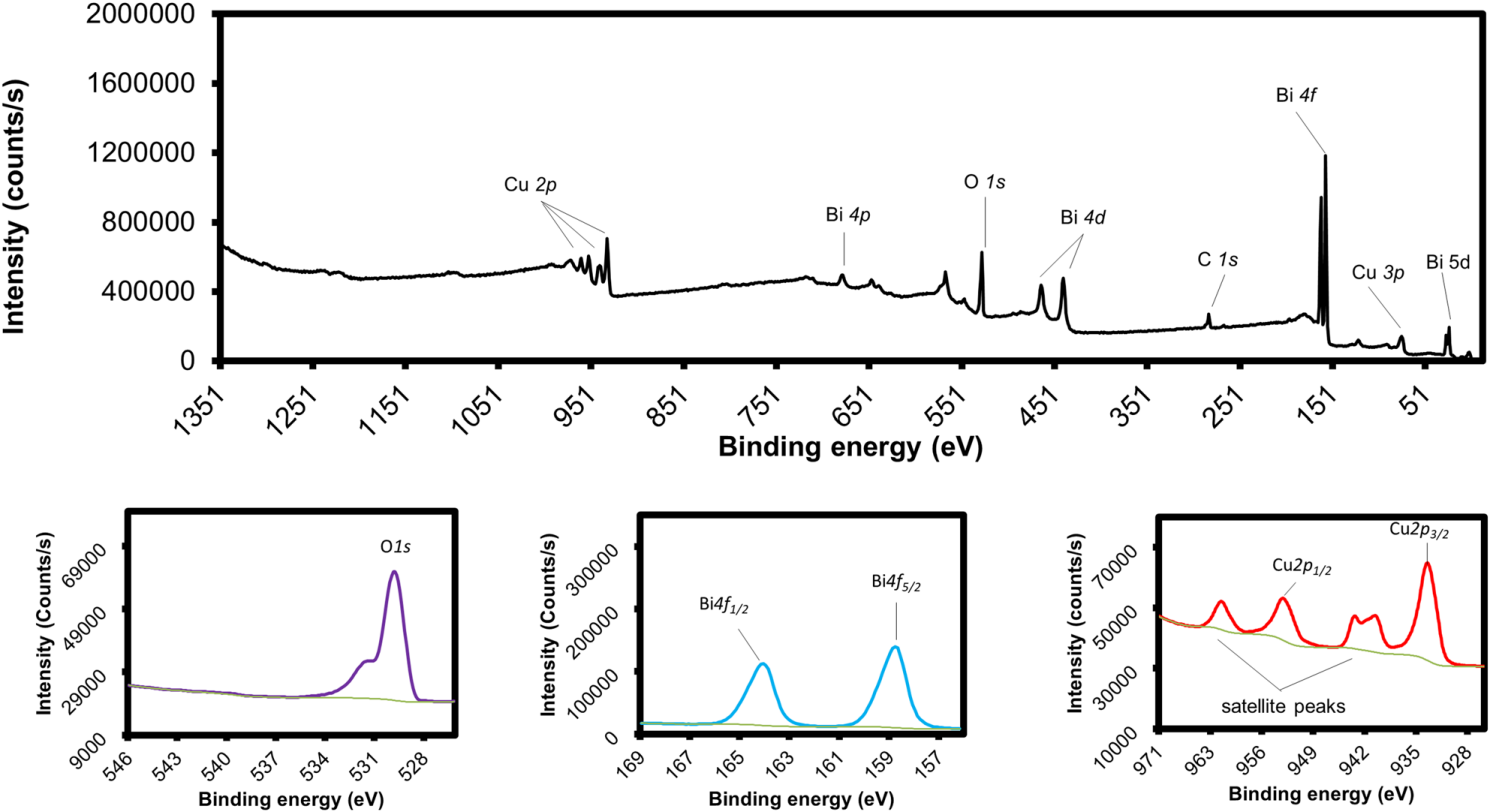
(**a**) X-ray photoelectron spectroscopy (XPS) survey spectrum of sample S15450 with the high-resolution XPS spectra of (**b**) O*1s*, (**c**) Bi*4f.*, and (**d**) Cu*2p.*

The aforementioned analyses indicated that S15450 may possess the necessary structure for improving the efficiency of PEC water splitting. The XPS technique was used to evaluate the chemical states of the elements on the surface of S15450. Fig. 3 shows the XPS spectrum of S15450. The survey spectrum shows that only three elements, i.e., Cu, Bi, and O (Fig. 3a) existed in the sample. The three high-resolution spectra of S15450 in Fig. 3b–d show the peaks of O, Bi, and Cu. The peak of element O*1s* at 529.65 eV was attributed to lattice O, while the small shoulder peak at 531.25 eV was ascribed to O absorption or defects^29^; Two Bi peaks originating from Bi*4f*_*3/2*_ and Bi*4f*_*1/2*_ were found at 158.57 and 163.88 eV, respectively, confirmed the presence of Bi^3^^+^ in CuBi_2_O_4_^29^. The XPS spectrum of Cu shows two main peaks at 933.51 and 953.69 eV that originated from Cu*2p*_*3/2*_ and Cu*2p*_*1/2*_, respectively. Moreover, the Cu*2p* peak positions and the appearance of two satellite peaks reveal the existence of Cu^2^^+^ in CuBi_2_O_4_^32^. Additionally, the XPS spectra (survey and high-resolution spectra) of all samples were acquired to investigate the effect of coating number and annealing temperature. The spectra are shown in SI–Fig. S6 and S7. Overall, all samples exhibited similar peaks in their spectra. The negligible changes in the XPS peaks of the elements with the variation in annealing temperature and thickness indicated the absence of change in the chemical environment of elements in the lattice^29^.

The ability of a material to absorb light is the most important factor in the water splitting via PEC. The material must efficiently absorb photons from the light source and generate e^−^ – h^+^ pairs to produce photo current density^17^. UV-visible spectrophotometry was performed to obtain an insight into the possibility of light harvesting by the FTO/CuO/CuBi_2_O_4_ photocathodes. The absorbance spectra of FTO/CuO/CuBi_2_O_4_ photocathodes are shown in SI–Fig. S8. The optical properties of any material are heavily dependent on the crystal microstructure^33^. All photocathodes exhibited strong absorption at around 300–400 nm, which subsequently reduced the incident photon with low energy (> 600 nm). S15450 and S10550 exhibited the highest absorption at 337 nm, indicating that FTO/CuO/CuBi_2_O_4_ photocathodes can only absorb the photons under high-energy illumination (violet part). These findings, in combination with morphological properties (Fig. 2), clearly illustrated the influence of morphology on optical properties. Samples with rough surfaces and irregular structures produce considerable light scattering, thereby reducing the perceived absorbance^34^. In particular, S05550, and S10550 had some voids in their surfaces, and S15550 contained some vertical structures that increased light scattering and reduced light absorption.

The Tauc plot of the photocathodes was obtained using Eq. (4) and the estimated bandgap was defined as follows:

4 $$\alpha hv = {\text{A}}\left( {hv - {\text{E}}_{{\text{g}}} } \right)^{{1/{\text{n}}}}$$

where n takes the value of ½ for the direct (allowed) bandgap Tauc plot; A denotes the absorption constant; and α, E_g_, and ν denote the absorption coefficient, bandgap, and light frequency, respectively^33^. The bandgaps of FTO/CuO/CuBi_2_O_4_ are presented in Table 1; these bandgaps were a slightly wider than those of the CuO thin film (2.12 eV)^28^. SI–Fig. S9 showed the bandgaps based on the absorption of FTO/CuO/CuBi_2_O_4_ under different experimental treatments with approximately similar light adsorption ranges calculated on the basis of (αhν)^2^ versus hν. A small change in bandgap energy can also cause a slight improvement in material absorptive capacity. Specifically, the sample with narrow bandgap of 1.51, 1.55, 1.65 and 1.74 eV includes S15450, S15550, S10550 and S10450, respectively. Wide bandgaps were found in samples in five coating layers samples (including samples S05350, S05450 and S05550 with bandgap of ~2.71, 2.66, and 2.45 eV respectively). The bandgaps of these photocathodes widened owing to the incomplete conversion of the coating to CuBi_2_O_4_ at 350 °C, as discussed earlier. In addition, compared in the same coating layer, higher temperature led to decrease bandgap. Because the crystal lattice tends to expands and the inter atomic bond is weaker at higher temperature. However, bandgap of sample S15550 is a little bit higher than S15450, it is probably due to the appearance of vertical structures on the surface. Previous studies^24,25,34^ have shown that CuBi_2_O_4_ and CuO can form the staggered band alignments owing to theirs type II heterojunction.

**Figure 4:**
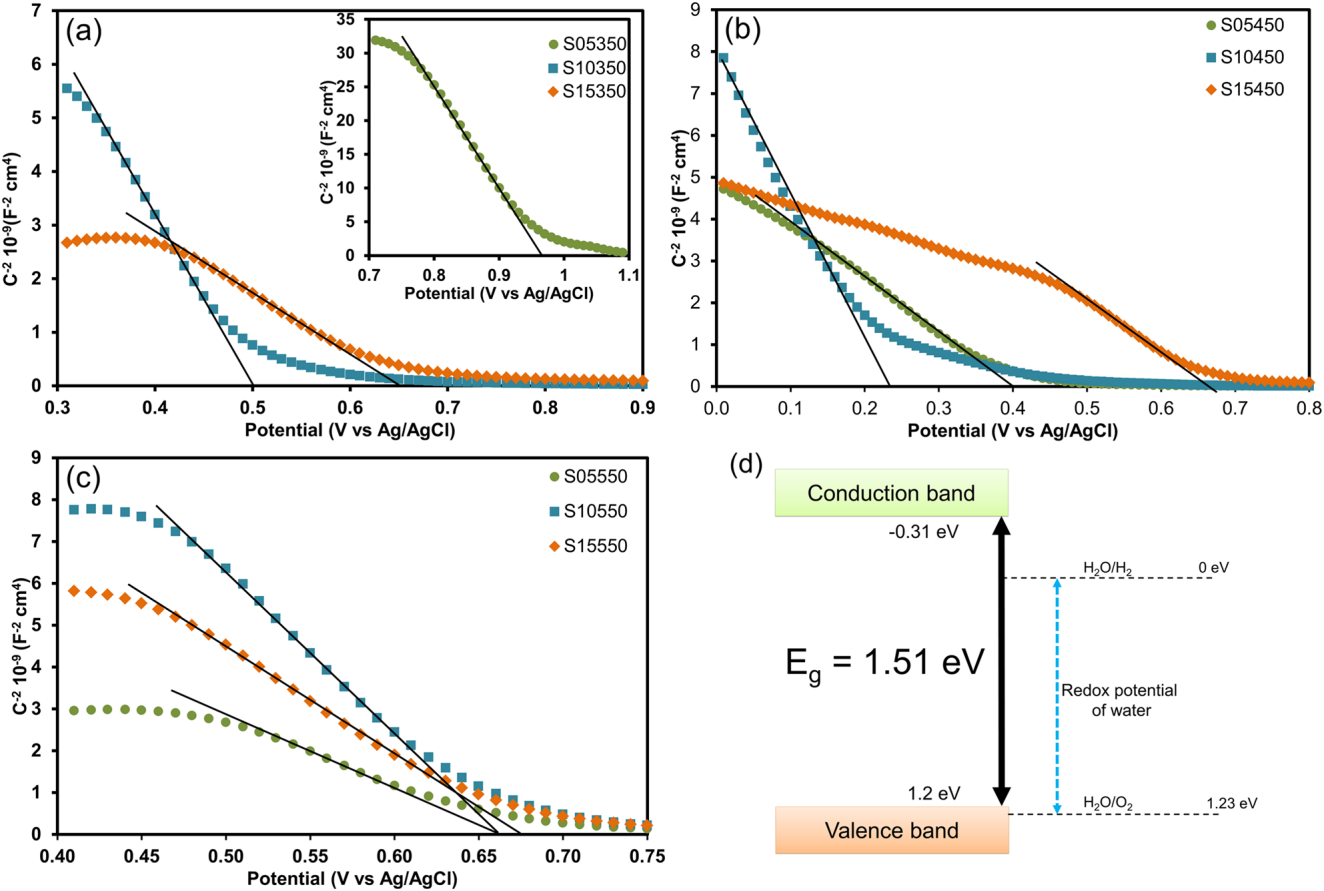
Mott-Schottky plots of FTO/CuO/CuBi_2_O_4_ photocathodes in a 0.1 M Na_2_SO_4_ electrolyte at 1 kHz. (**a**) photocathodes annealed at 350 °C (the inset shows the plot of S05350); (**b**) photocathodes annealed at 450 °C ; (**c**) photocathodes annealed at 550 °C; and (**d**) the proposed band position scheme of the S15450 photocathode compared with the redox potential of water.

Mott-Schottky analysis was performed at 1 kHz in a 0.1 M Na_2_SO_4_ aqueous solution to estimate the flat-band of the FTO/CuO/CuBi_2_O_4_ photocathodes. The results are presented in Table 1. The change in the flat-band potential of the materials under different experimental treatments (see Fig. 4a–c) indicated that the regression line of the FTO/CuO/CuBi_2_O_4_ photocathodes had a negative slope, indicating that the photocathodes were p-type semiconductors. Semiconductor S15450 exhibited a flat-band value of 0.67 V versus Ag/AgCl (1.2 V vs. RHE). The flat-band potential of a p-type semiconductor is roughly equal to that of its valence band^35,36^. As inferred from this result and SI–Fig. S9, the conduction band of the sample is located at − 0.31 V versus RHE. The band position of the electrode (proposed demonstration sample S15450) is shown in Fig. 4d. The bandgap precisely straddles in the redox potential of water demonstrating the potential of the FTO/CuO/CuBi_2_O_4_ photocathode to generate hydrogen via water splitting^36^.

**Figure 5:**
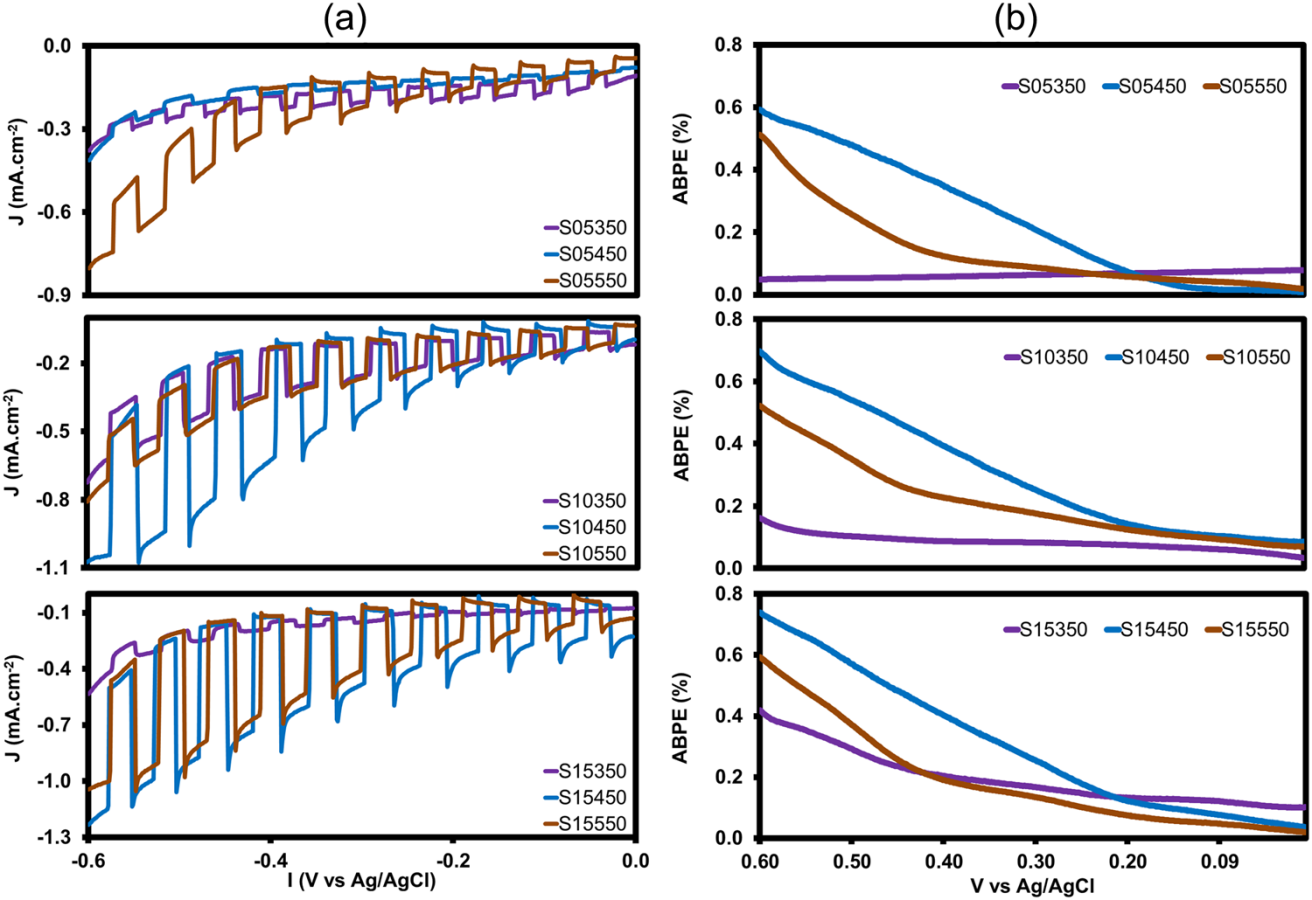
(**a**) Comparison of current density (chopped linear scanning voltammetry (LSV)) in a 0.1 M Na_2_SO_4_ electrolyte solution at pH 6.89 under illumination using Xe lamp (AM1.5G 100 mW/cm^2^); (**b**) Applied bias photo-to-current efficiencies (ABPEs) of FTO/CuO/CuBi_2_O_4_ photocathodes obtained from three-electrode configuration calculated and plotted from LSV data (Eq. 8).

PEC experiments were conducted in a three-electrode configuration. FTO/CuO/CuBi_2_O_4_ with various numbers of coating layers and heat treatment was used as the photocathode. The current densities are measured at − 0.6 V versus Ag/AgCl at a scan rate of 10 mV/s. The chopped (dark/light) linear scanning voltammetry (LSV) scans of the CuO, 5CL450, and 5CL layers are presented in SI–Fig. S10. As can be seen, the CuO layer exhibited a potential of 0.49 mA/cm^2^ under the applied voltage of − 0.6 V versus Ag/AgCl, whereas the 5CL450 layer exhibited a slightly higher potential (0.36 mA/cm^2^) under the same applied potential. The coating layers that were not subjected to annealing did not react to light and their potential reached 0.27mA/cm^2^. Another experiment was conducted to compare the PEC performances of the CuO and CuBi_2_O_4_ layers combined into a heterojunction. The results demonstrated at the current density increased sharply (Fig. 5a). In the heterostructure, the CuO layer of the photocathode served as an electron donor in the PEC process and the CuBi_2_O_4_ film acted as a protective layer against the photocorrosion of the underlying CuO layer and as the transport layer that transferred electrons to the electrode/electrolyte interface^24^. Among all photocathodes in all coating and temperature cases FTO/CuO/CuBi_2_O_4_ at annealed 450 °C exhibited the highest current density. Specifically, the current densities of S10450 and S15450 were 1.07 and 1.23 mA/cm^2^ at − 0.6 V_Ag/AgCl_, respectively. The lowest efficiency was exhibited by S05350, S10350, and S15350; the corresponding current densities were 0.38, 0.72, and 0.53 mA/cm^2^ at − 0.6 V_Ag/AgCl_, respectively. These results were consistent with the analysis of bandgap structure presented earlier. The enhanced current density of FTO/CuO/CuBi_2_O_4_ annealed at 450 °C had two possible reasons: First, the improvement in crystal size led to improved charge transport in layers with reduced grain boundaries. Second, CuBi_2_O_4_ completely crystallized at 450 °C, the coating was not completely converted at 350 °C, and CuBi_2_O_4_ had an unstable structure, and formed voids on its surface when annealed at 550 °C (discussed in the SEM section).

The applied bias photo-to-current efficiencies (ABPEs) of the samples are presented in Fig. 5b. APBE is used to quantitatively evaluate the hydrogen PEC generation efficiency of photocathodes^34,37^ and was calculated on the basic of the applied bias between the counter and working electrode and LSV measurements (Eq. 8). Comparing the three annealing temperature levels revealed that the photocathodes fabricated at 450 °C exhibited a marked enhancement in efficiency compared with the samples annealed at 350 °C. Additionally, annealing at 550 °C caused a reduction in ABPE owing to the formation of a heterogeneous structure (see SI–Fig. S3dgj)^37^. In the group of photocathodes annealed at 450 °C, the sample with 15 coating layers achieved the highest efficiency (0.74%) compared with the samples with 10 and 5 coating layers (0.69% and 0.59%, respectively). These results demonstrated that the photocathode was effective in splitting e^−^–h^+^ pairs and can inhibit charge carrier recombination, thereby improving the overall efficiency of PEC water splitting^38^.

**Figure 6:**
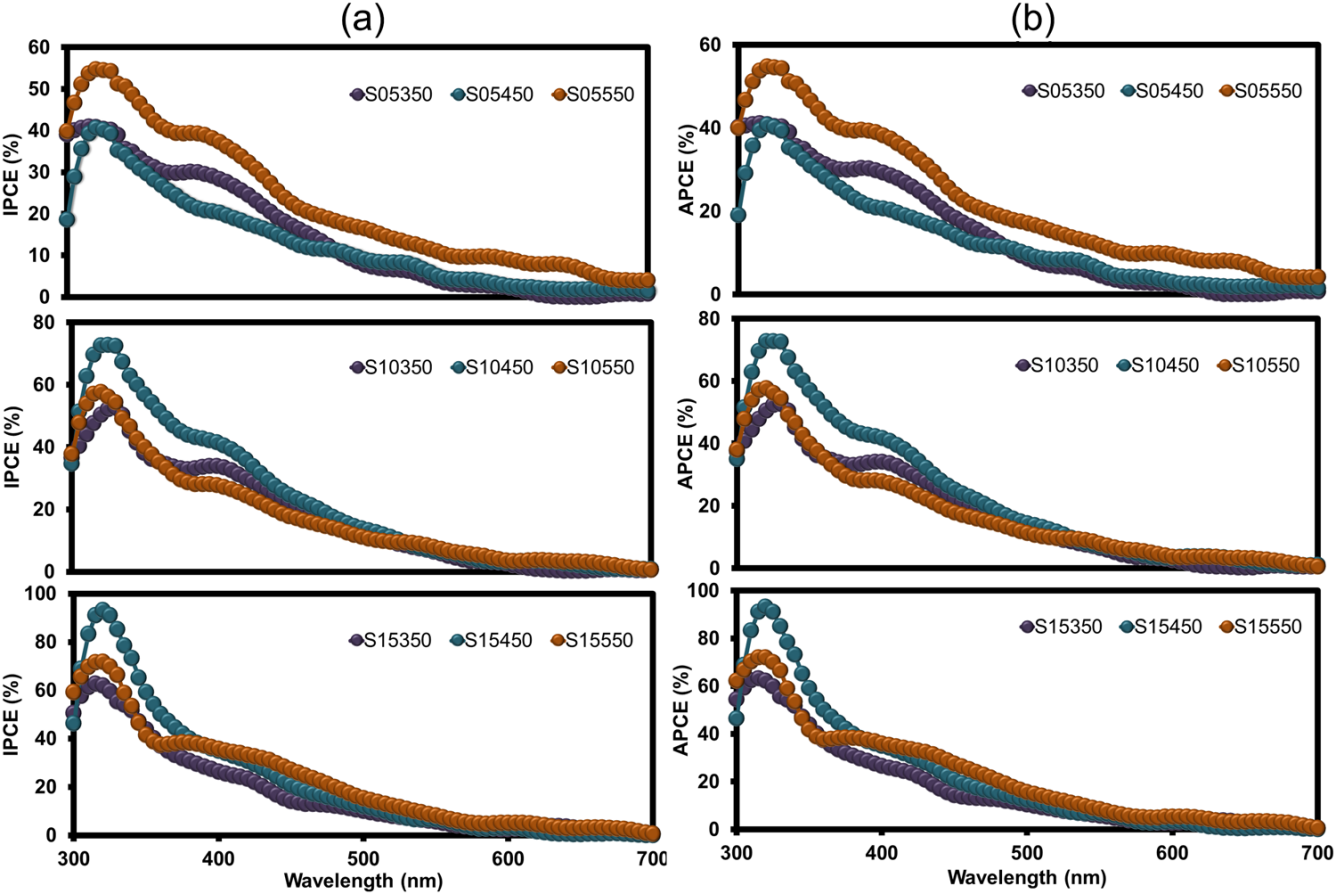
(**a**) Incident photon-to-current conversion efficiency (IPCE) plot of FTO/CuO/CuBi_2_O_4_ fabricated with different number of coating layers and at different annealing temperatures. IPCE was measured at a potential of − 0.6 V in a 0.1 M Na_2_SO_4_ and calculated using the Eq. (9); and (**b**) Absorbed photo-to-current density (APCE) plot obtained from IPCE data and absorbance values (Eq. 10).

Incident photon-to-current conversion efficiency (IPCE) was used to measure the ratio of photocurrent versus incident photons as a function of wavelength to elucidate the mechanism of the FTO/CuO/CuBi_2_O_4_ photocathode^34^ (Fig. 6a). As expected, ICPE tended to be similar to the measured current density. IPCE peaked at approximately 310–340 nm and increased with the increasing number of coating layers; the highest conversion efficiency was exhibited by S15450 (93.14%). All samples exhibited low external quantum efficiencies at long-wavelength (> 600 nm) because they had low quantum yield in the conversion of with energies close to the bandgap^16^. The IPCE data were used to calculate the absorbed photo-to-current density (APCE) by Eq. (10). FTO/CuO/CuBi_2_O_4_ can convert most of the photons close to the absorption edge as proven by measuring the bandgap and absorption of the photocathodes. The APCE curves of all photocathodes were similar in shape, and APCE values were not significantly higher than IPCE values (Fig. 6b). Peaks were located at approximately 310–340 nm (with 93.16%), and the internal quantum efficiencies decreased to zero at long wavelengths (>600 nm). The differing APCE values of the photocathodes and the lower APCE indicate of differing absorption or the poor transport of carriers^39^. The IPCE and APCE of S15450 were 11.98 and 8.91 times higher than the external and internal quantum efficiencies of the CuO layer, respectively (SI–Fig. S11). These findings illustrate that the CuBi_2_O_4_ layer exhibited good efficiency in absorbing photons and transferring charge carriers to generate photocurrents. As illustrated in Fig. 6, and SI–Fig. S11, all the photocathodes exhibited good transition in the violet part of illumination (300–400 nm), indicating that only incident light with high energy may be absorbed and converted to currents.

**Figure 7:**
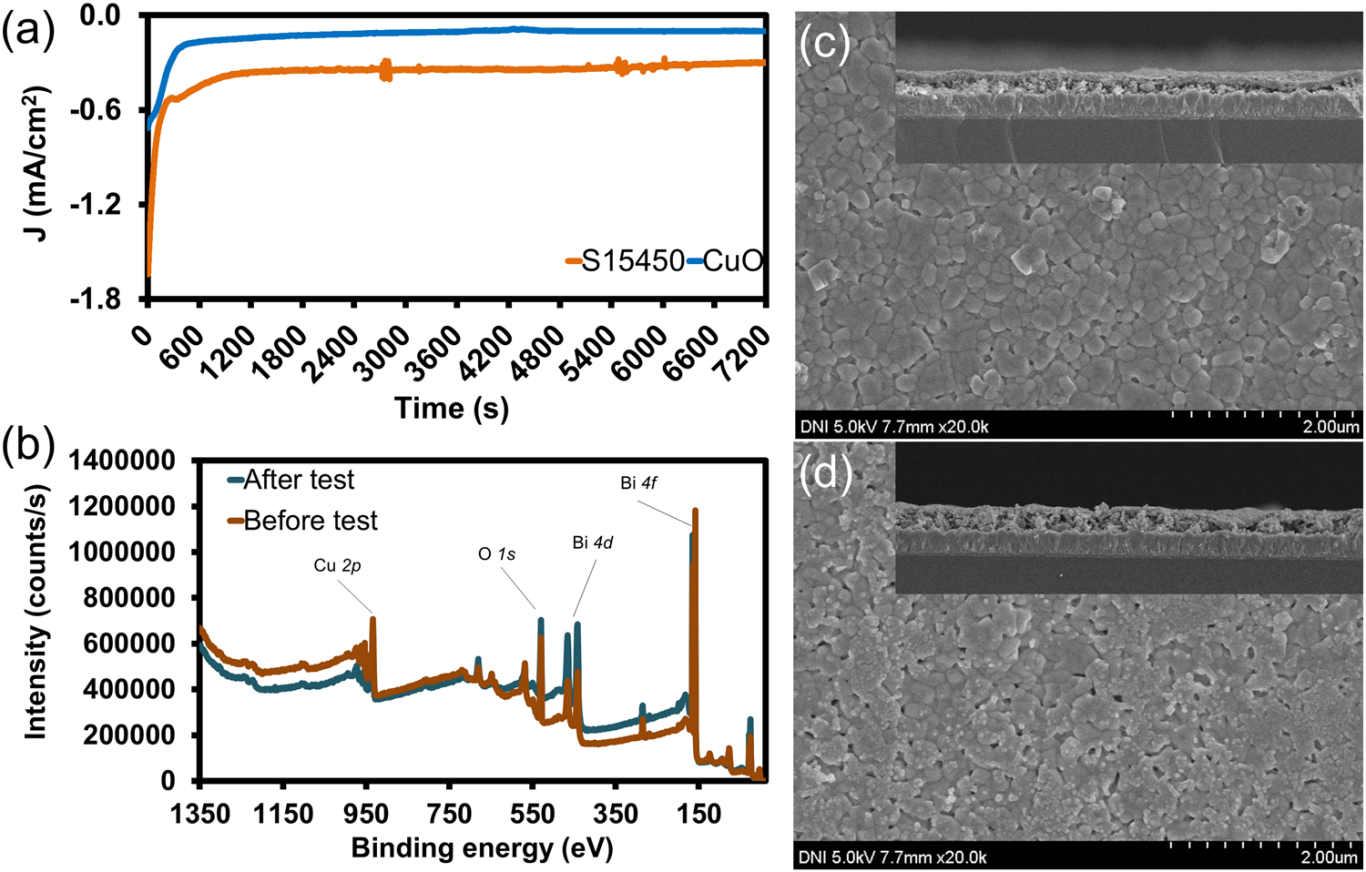
(**a**) Stability test on FTO/CuO and S15450 (FTO/CuO/CuBi_2_O_4_) photocathodes under 7200 s of illumination (0.1 M Na_2_SO_4_ electrolyte pH 6.8). The test performed at an applied potential of -0.6 V versus Ag/AgCl; (**b**) the XPS survey spectrum of sample S15450 before and after stability test; (**c**) and (**d**) are the SEM images (the inset images are the cross-section of each sample) of photocathode S15450 before and after stability test, respectively;

A chronoamperometry test was conducted on FTO/CuO and FTO/CuO/CuBi_2_O_4_ (S15450) in a 0.1 M Na_2_SO_4_ electrolyte (pH 6.8) at a fixed potential (− 0.6 V vs. Ag/AgCl) under continuous illumination for 7200 sec to test the PEC stability of the photocathodes; the results are presented in Fig. 7a. The *J–t* plot shows that the current density of the photocathode coated with CuBi_2_O_4_ increased. Consistent with the data in Fig. 5, the photocurrent of the photocathode with CuBi_2_O_4_ layer shows two times higher than that of the photocathode without CuBi_2_O_4_. The photocurrent density of both photocathodes decreased rapidly at first ~300 s and then gradually stabilized during the stability test, indicating that the semiconductor-electrolyte interface was affected initially^40^; after a period of adaptation with medium, the photocathode gradually stabilizes. Morphology change of photocathode S15450 is given in Fig. 7c, d. Apparently after applying external voltage (− 0.6 V vs. Ag/AgCl) and reacting in the electrolyte for 7200 s, the surface of electrode have changed a little bit. Specifically, CuBi_2_O_4_ layer was corroded at some points. The cross-section exhibited the present of CuO/CuBi_2_O_4_ layer on the top, but thinner. After the stability test in neutral electrolyte, the XPS spectrum (Fig. 7b) exhibited the profile of the O*1s*, Cu*2p*, and Bi*4f* peaks is still similar to those of sample before test. Normally, the reaction on the photocathode’s surface is the reduction, which will attract positive ions (H^+^) to the interface and reduce to H_2_ gas, or the material of photocathode will be reduced to other substances. Since there is no change, i.e. the peaks have not been shifted, on the XPS spectra between before and after the test, the CuO/CuBi_2_O_4_ photocathode has not been converted to another substance but is slightly corroded on the surface. Compared to the result of stability test, surface corrosion can occur in the first ~300 s, so it led to a significantly reduced current density. After that, it would have been possible that less (or no further) corrosion would occur, due to the steady generated current density during the rest of the stability test. The FTO/CuO photocathode showed a relatively low PEC efficiency that improved when it was coated with CuBi_2_O_4_ without the loading of other precious metals. Therefore, CuBi_2_O_4_ has potential to increase the efficiency of photocathodes. To further elucidate the role of CuBi_2_O_4_ layer in increasing the efficiency of current density of CuO/CuBi_2_O_4_ photocathode, PL spectra were investigated. Fig. S12 exhibited the PL intensity of CuBi_2_O_4_ layer was quite low, and this spectrum matched with the report of Wang et al.^41^. The similar PL peaks of CuO and CuO/CuBi_2_O_4_ show that the CuBi_2_O_4_ thin layer does not compete with CuO^42^. In addition, the PL emission intensity of the CuO layer decreased sharply after the inclusion of CuBi_2_O_4_, demonstrating that the carriers are separated more efficiently due to the presence of type II heterojunctions with suitable high-energy band sites^25^. The PL emission peaks from the CuO/CuBi_2_O_4_ photocathode become relatively weak, indicating a decrease in electron-hole recombination^41^. As can be clearly determined from these results, CuBi_2_O_4_ acts as a protective layer to prevent self-reduction of the CuO layer, increase photon absorption, as well as limit recombination of e^−^–h^+^ pair by transporting electrons accumulated on the edge of the CuO layer to the electrode/electrolyte interface and perform the reduction reaction of H^+^ to H_2_. Table 2 presents the comparison of the stabilities reported by other studies on CuBi_2_O_4_. In contrast to the photocathodes reported by other studies, the FTO/CuO/CuBi_2_O_4_ photocathode developed in this study exhibited good and stable PEC performance in the water splitting process without the addition of rare metals.

**Table 2:** Comparing of the photoelectrochemical stabilities of CuBi_2_O_4_-related photocathodes.

Photocathode	Applied Potential	Electrolyte (pH)	Testing time (s)	Photocurrent density (at stable period) (mA/cm^2^)	Ref.
FTO/CuO/CuBi_2_O_4_	− 0.6 V versus Ag/AgCl	0.1 M Na_2_SO_4_ (pH 6.7)	7200	0.4	In this study
CuO/CuBi_2_O_4_/NiO_x_	0.6 V versus RHE	0.1 M NaOH (pH = 12.8)	18,000	~ 0.5	^26^
CuBi_2_O_4_	0.6 V versus RHE	0.3 M K_2_SO_4_ and 0.2 M phosphate buffer (pH 6.65) (With H_2_O_2_ as an electron scavenger)	7200	~ 0.97	^17^
CuBi_2_O_4_/ZnSe/P25	0.3 V versus RHE	0.3 M K_2_SO_4_ and 0.2 M phosphate buffer (pH = 6.65)	5000	0.43	^38^
Bare CuBi_2_O_4_	0.6 V versus RHE	0.3 M K_2_SO_4_ and 0.2 M phosphate buffer (pH 7)	18,000	~ 0.1	^39^
CuBi_2_O_4_	− 0.4 V versus Ag/AgCl	0.1 M Na_2_SO_4_	200	0.45	^43^
CuBi_2_O_4_/Au/N, Cu–C	0.5 V versus RHE	0.3 M K_2_SO_4_ and 0.2 M Phosphate buffer (pH 6.68)	3000	0.31	^44^

## Conclusion

A FTO/CuO/CuBi_2_O_4_ photocathode was synthesized using the hydrothermal method followed by spin coating and annealing. The techniques used in this study confirmed that 15 coating layers annealed at 450 °C formed a homogenous CuBi_2_O_4_ layer without cracking, or voiding on the surface and with a continuous interaction with the underlying CuO layer. The absence of gaps between the two layers supported charge transportation. The experimental results indicated that the optimal current density of 1.23 mA/cm^2^ was achieved at − 0.6 V_Ag/AgCl_ in a neutral electrolyte 0.1 M Na_2_SO_4_. The stability of the FTO/CuO/CuBi_2_O_4 _photocathode was better than that of the photocathode without CuBi_2_O_4_. These results confirmed the potential of the CuBi_2_O_4_ layer as a protective electrode layer that reduces recombination, improves the efficiency of the underlying CuO layer during PEC water splitting, and increases the current density. The intimate contact between CuO and CuBi_2_O_4_ can enhance the efficiency of PEC through the following mechanisms: (1) The protective function of the CuBi_2_O_4_ layer prevents the contact between the electrolyte and CuO layer, thereby reducing the potential of CuO for self-reduction into Cu_2_O or Cu in the PEC water splitting process; (2) The CuBi_2_O_4_ layer transports the electrons that have accumulated on the edge of the CuO layer to the electrode/electrolyte interface, thereby reducing recombination. This study demonstrated that when properly designed, p-type heterojunction materials can exhibit improved properties for HER.

## Methods

### Synthesis the CuO thin film

The CuO thin film was synthesized by the hydrothermal method^28^. First, the FTO substrates were cleaned in three steps using methanol, ethanol, and deionized water (DIW), respectively. The substrates were dried under a robust stream of nitrogen. A mixed precursor solution consisting of Cu(C_2_H_3_O_2_)_2_·H_2_O 0.5 M and CH_3_COONa 2 M was sonicated at 30 min before adding to a Teflon-lined stainless steel autoclave. The substrates (1.5 × 2 cm) were slanted inside the autoclave, the FTO side facing down. Next, the autoclaves were tightly covered and maintained at 150 °C for 12 h, with a heating rate of 1 h. After incubation, the samples were cleaned twice with DIW and ethanol, respectively. Finally, the CuO-coated substrate was dried on a hot plate at 60 °C for 30 min. All chemicals in this study were purchased commercially from Sigma Aldrich Company without further purification.

### Synthesis the CuBi_2_O_4_ layer

0.25 M Cu(NO_3_)_2_·3H_2_O was prepared in ethanol and 2 M Bi(NO_3_)_3_·5H_2_O was prepared in lactic acid. Each solution was stirred with a magnetic stirrer until completely dissolved. The precursor solution was prepared by mixing the above solutions (4 parts of Cu^2+^ and 1 part of Bi^3+^ to total volume) and a Cu:Bi atomic ratio of 1:2. The CuBi_2_O_4_ layer was synthesized by the spin coating method followed by heat treatment. Specifically, the above CuO samples were put on the spin coating machine, set the program at 2000 rpm for 20 s each time, and the precursor solution was dropped on the surface. Samples prepared for 5, 10 and 15 layers were dried for 10 min at 85 °C on a hot plate. Then the samples were incubated in the air for 2 h at 350 °C, 450 °C, and 550 °C. The experimental summary diagram has been shown in Figure S1.

### Characterizations

In order to confirm the characteristics of samples, 4 techniques were used. The Scanning Electron Microscope (SEM) technique was performed in Hitachi S-4800 model equipped with EDS was used to define the morphology and element composition of samples. The top view was taken to define the morphology changing of sample under different annealing temperature; the cross-section was taken for layer thickness determining. X-ray electron spectroscopy (XPS, ESCALABTM 250Xi model, Thermo ScientificTM) was used to determine the valence state as well as the chemical composition of the sample. PL measurements (Model: FluoroLog-iHR320, Horiba Jobin-Yvon) were performed at room temperature using a spectrophotometer with a 150 W Xenon lamp to verify the formation of heterojunction. The crystal and phase structure of samples were determined by XRD technique (using PANalytical X' pert PRO machine with Cu Kα radiation, λ = 1.5406 Å). From the XRD spectra, some structural properties were calculated including crystalline size (D), dislocation density (δ), and micro-strain (ε) using the Eqs. (5, 6, 7), respectively^28^.

5 $${\text{D}} = \frac{{{\text{k}}\lambda }}{{{{\upbeta }} \cdot \cos {{\uptheta }}}}$$

6 $${\updelta } = { }\frac{1}{{{\text{D}}^{2} }}$$

7 $${\upvarepsilon } = { }\frac{{\upbeta }}{{4 \cdot \tan {\uptheta }}}$$

where k = 0.94 which is the Scherer constant, λ = 1.54178 Å which is the wavelength of X-ray, β is the full width at half maximum (FWHM) of the diffraction peak and θ is the Bragg’s angle of diffraction peak.

### Photoelectrochemical measurements

PEC measurements were performed in a three-electrode configuration consisting of a working electrode (samples), a reference electrode (Ag/AgCl) and a counter electrode (Pt plate). Samples were applied with Loctite 1C Hi-Sole Epoxy/White adhesive for electrode connection as well as limiting the remaining active area to 1 cm^2^. Electrolyte was 0.1 M sodium sulfate buffer (pH = 6.89). A Xe illuminator (AM1.5G 100 mW/cm^2^) was used to simulate the light source in this experiment. Photo-current density was obtained by applying a linear sweep voltammetry (LSV) with a scan rate of 10 mV/s in the voltage range − 0.6 to 0.0 V. Besides, there are some diagnostics in the PEC measurement were used in this study. The Applied Bias Photo-to-current Efficiency (ABPE) was calculated by Eq. (8)^34^. Eq. (9) and (10) were used to calculate the Incident Photo-to-current Efficiency (IPCE) and Absorb Photo-to-current Efficiency (APCE), respectively^34^.

8 $${\text{ABPE}} = { }\frac{{{\text{J}}_{{{\text{ph}}}} { } \times \left( {1.23 - {\text{V}}} \right)}}{{{\text{P}}_{{{\text{light}}}} }} \times 100{\text{ \% }}$$

9 $${\text{IPCE}} = \frac{{\left| {{\text{J}}_{{{\text{ph}}}} } \right| \times {\text{hc}}}}{{{\text{P}}_{{{\text{mono}}}} \times \lambda }}$$

10 $${\text{APCE}} = { }\frac{{{\text{IPCE}}}}{{{\upeta }_{{{\text{e}}^{ - } /{\text{h}}^{ + } }} }}$$

where J_ph_ (mA/cm^2^) is the measured photocurrent density, P_mono_ is monochromatic illuminance power density, c is the speed of light, h is a constant of Planck, λ is the wavelength, ${\upeta }_{{{\text{e}}^{ - } /{\text{h}}^{ + } }}$ is the absorbance, P_light_ (mW/cm^2^) is the incident light power density, and V is the potential applied in the cell (vs. Ag/AgCl). The applied potentials in all experiments were converted to the reversible hydrogen electrode (RHE) scale using the Nernst equation (Eq. 11)^45^:

11 $${\text{E}}_{{{\text{RHE}}}} = {\text{ E}}_{{\left( {{\text{Ag}}/{\text{AgCl}}} \right)}} + {\text{ E}}_{{\left( {{\text{Ag}}/{\text{AgCl}}} \right)}}^{0} + 0.059{ } \times {\text{pH}}$$

where E_(Ag/AgCl)_ is the potential applied on working electrode, E^o^_(Ag/AgCl)_ = 0.197 V at ambient temperature (25 °C).

## Supplementary Information

Supplementary Figures.

## Data Availability

The data supporting the findings of this research are available within the article and supplementary information.
