# Supplementary material for: Improving the photoelectrochemical water splitting performance of CuO photocathodes using a protective CuBi2O4 layer
Source: Sci Rep. 2023 Apr 8;13:5776. doi: 10.1038/s41598-023-32804-0 (PMC10082760; doi:10.1038/s41598-023-32804-0)
Supplement: Supplementary file 1 — Supplementary Figures. [file 41598_2023_32804_MOESM1_ESM.docx]

**SUPPLEMENTATY INFORMATION**

Improving the photoelectrochemical water splitting performance of CuO photocathode by using the CuBi_2_O_4_ protective layer

# Nguyen Hoang Lam^1^, Nguyen Tam Nguyen Truong^1*^, Nam Le^1^, Kwang-Soon Ahn^1^, Younjung Jo^2^, Chang-Duk Kim^2*^, and Jae Hak Jung^1*^

^1^ School of Chemical Engineering, Yeungnam University, 280 Daehak-Ro, Gyeongsan 38541, Republic of Korea;

^2^ Department of Physics, Kyungpook National University, Daegu, 702-701, Republic of Korea.

*corresponding authors: [tamnguyentn@ynu.ac.kr](mailto:tamnguyentn@ynu.ac.kr) (N.T.N.T), [duks@knu.ac.kr](mailto:duks@knu.ac.kr) (C.D.K) and [jhjung@ynu.ac.kr](mailto:jhjung@ynu.ac.kr) (J.H.J)


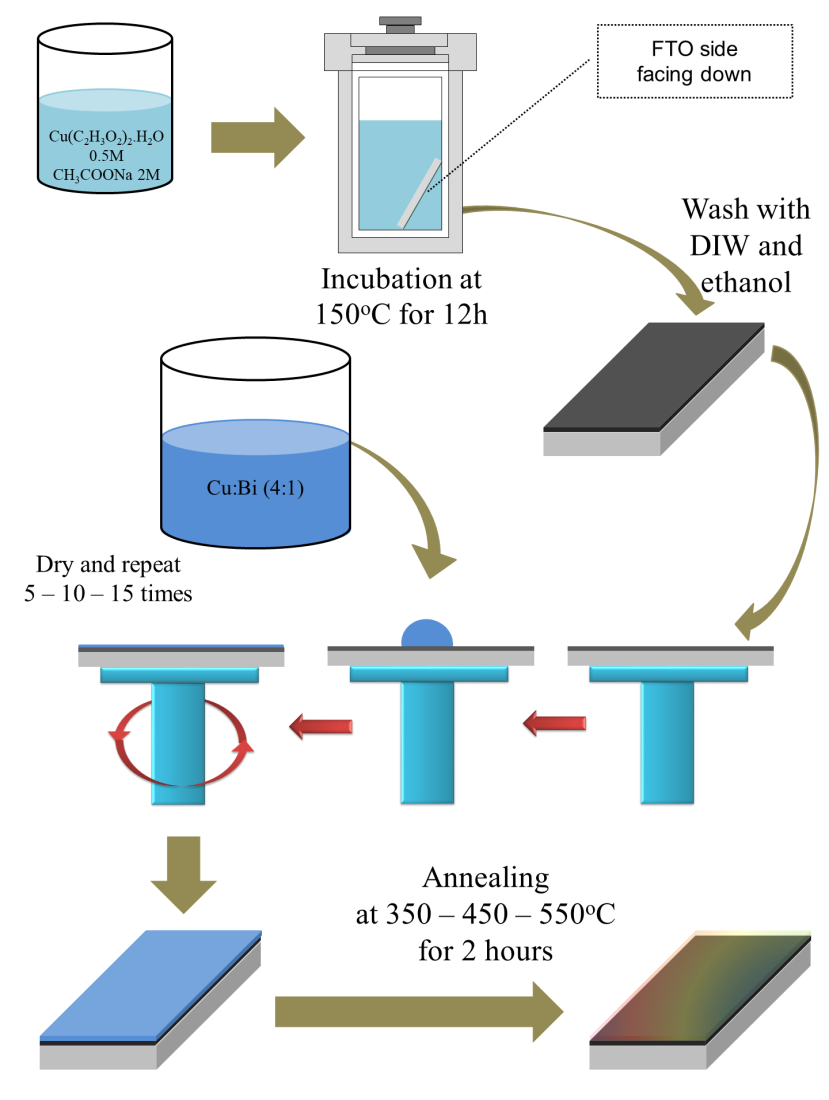


**Figure S1.** The experimental summary diagram


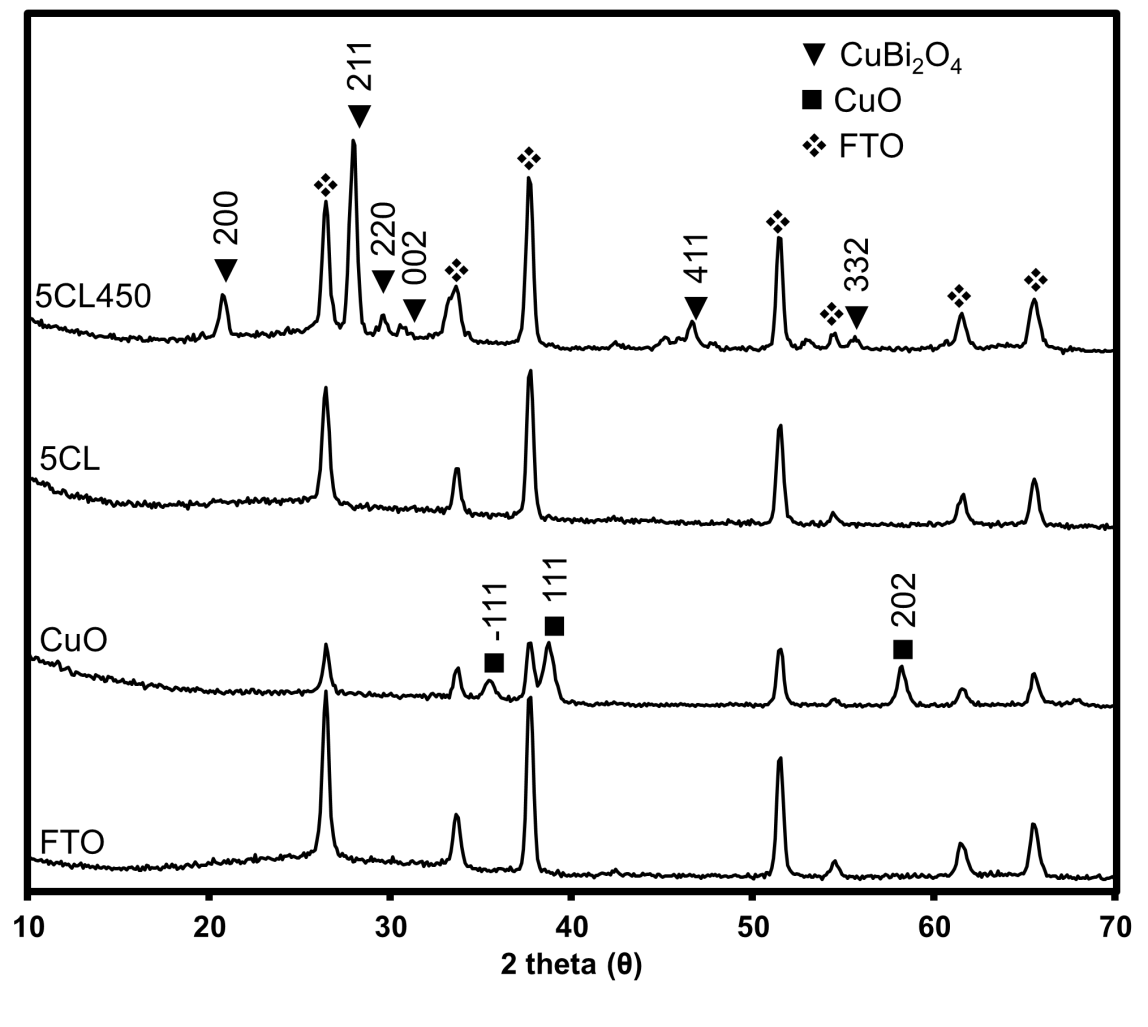


**Figure S2.** XRD spectrum of CuO thin film; CuBi_2_O_4_ before and after annealing (5 coats with annealing temperature of 450 °C) on FTO substrate. The blue square dots represent the peaks of CuO crystals (ICDD No#00-041-0254), the red triangular dots represent the peaks of CuBi_2_O_4_ crystals (JCPDS No.42.0334)


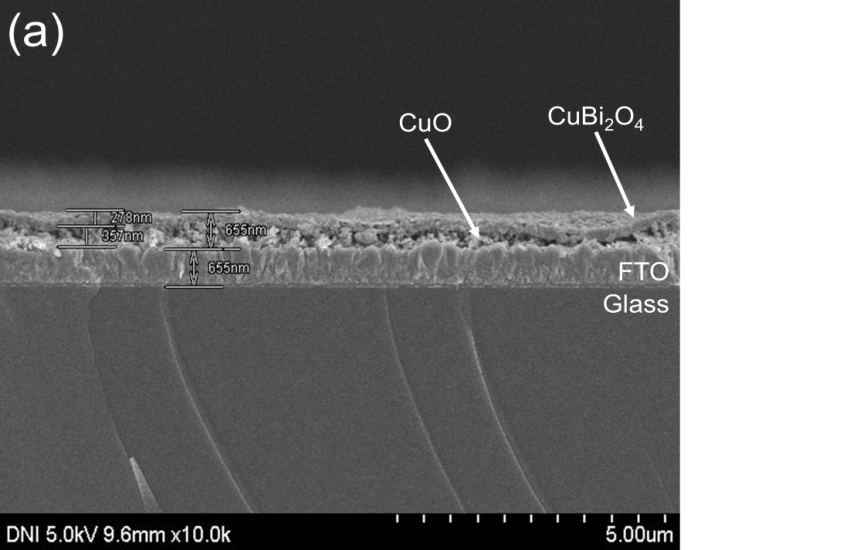

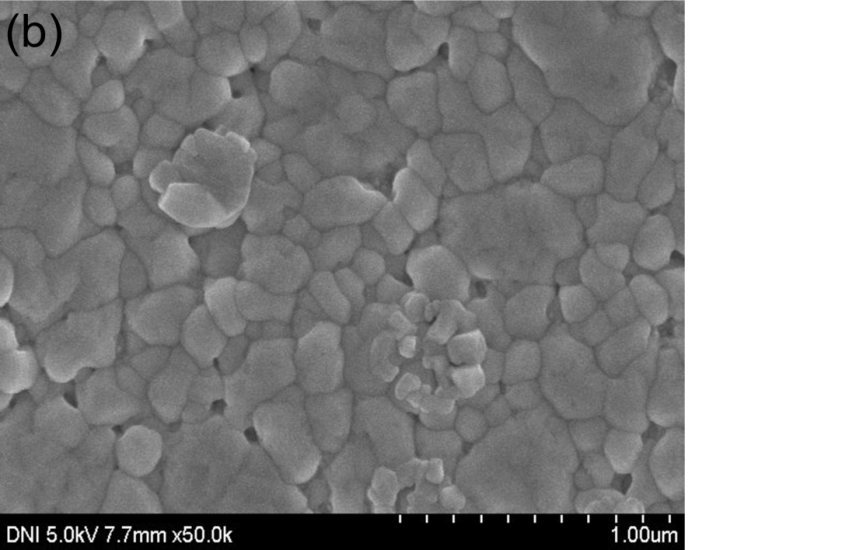


**Figure S3.** (a) Cross-section view and (b) plane view SEM image of FTO/CuO/CuBi_2_O_4_ with 15 coating layers and annealing at 450 ^o^C (sample S15450)


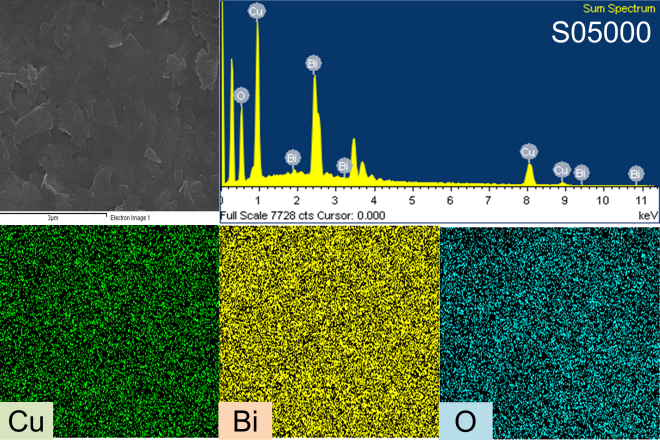

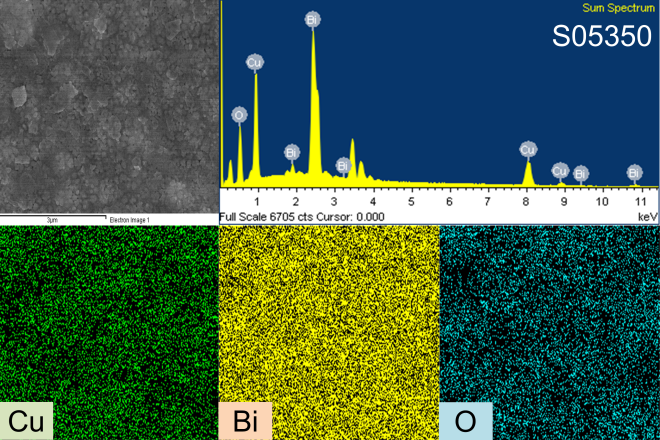


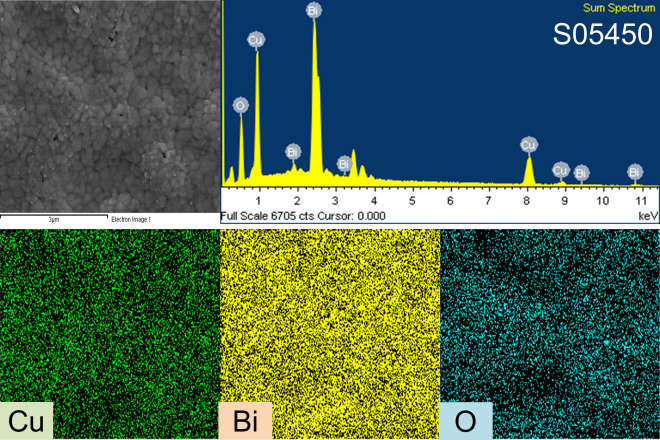

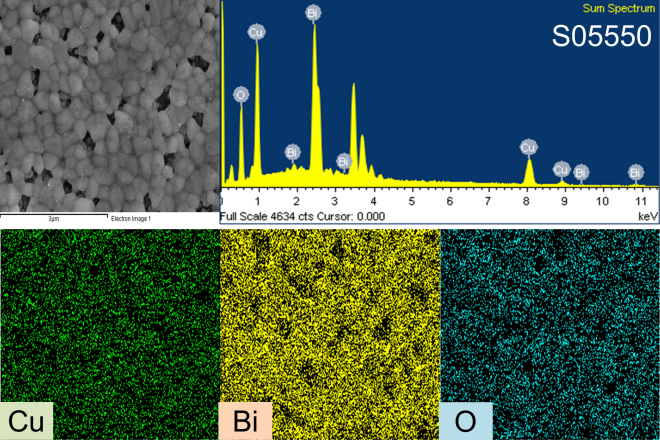


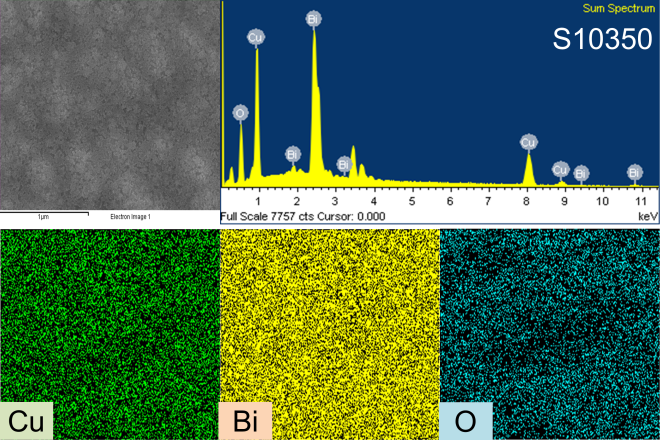

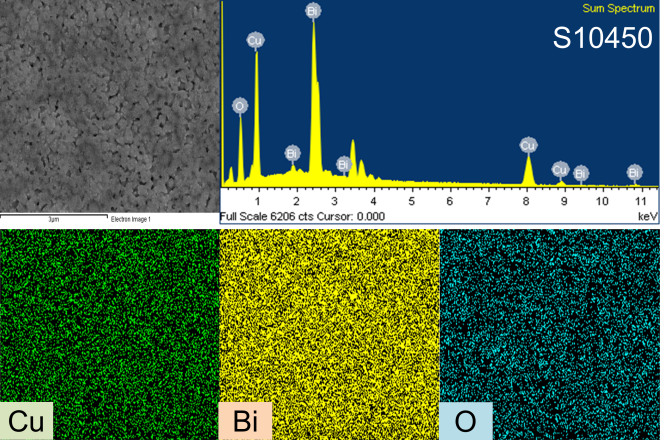


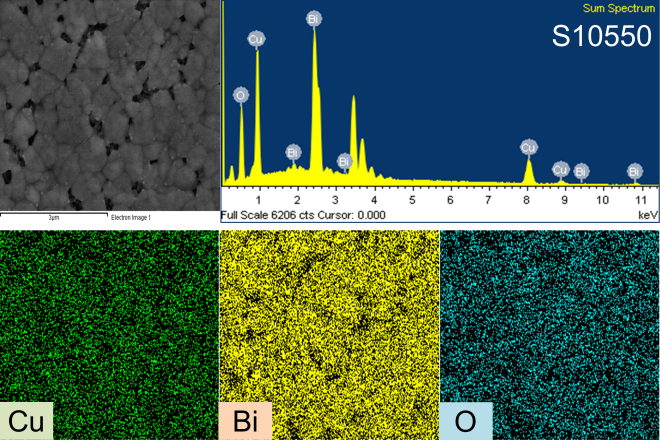

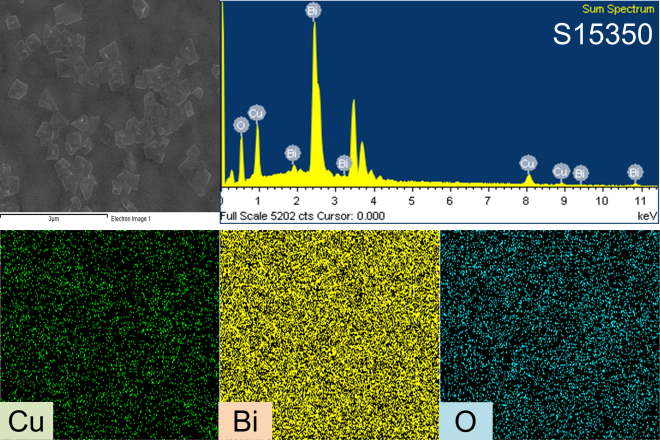


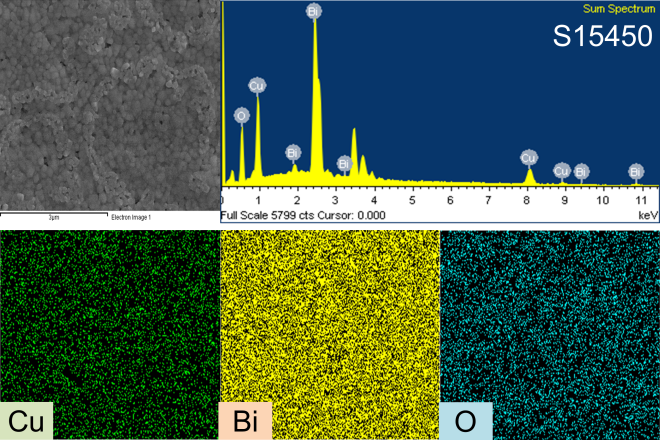

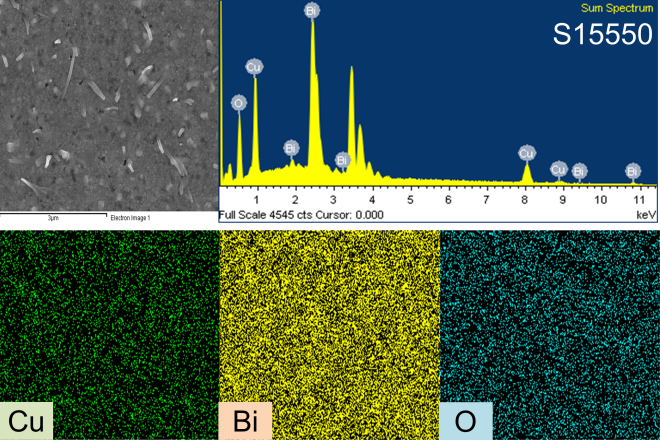


**Figure S4.** Top-down SEM-EDS mapping image of FTO/CuO/CuBi_2_O_4_ photocathode. Each sample has 5 images; the top-left image is the EDS study area; the figure on the top-right is a histogram of the elements; the elements Cu, Bi, and O are shown in the three below figures, shown on the left - in green, in the middle - in yellow, and on the right - in blue, respectively.


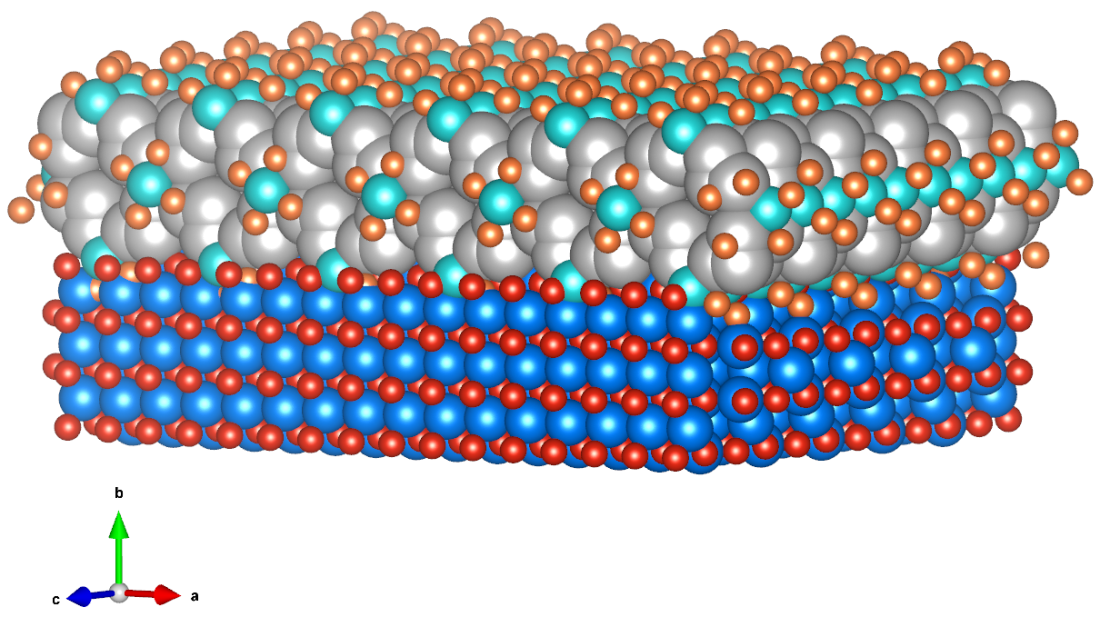


**Figure S5.** The heterojunction can be defined and modelled by VESTA software (version 3). Blue dots are Cu element in CuO, red dots are O element in CuO; Grey, cyan, and orange dots are Cu, Bi, and O elements in CuBi_2_O_4_ structure, respectively.


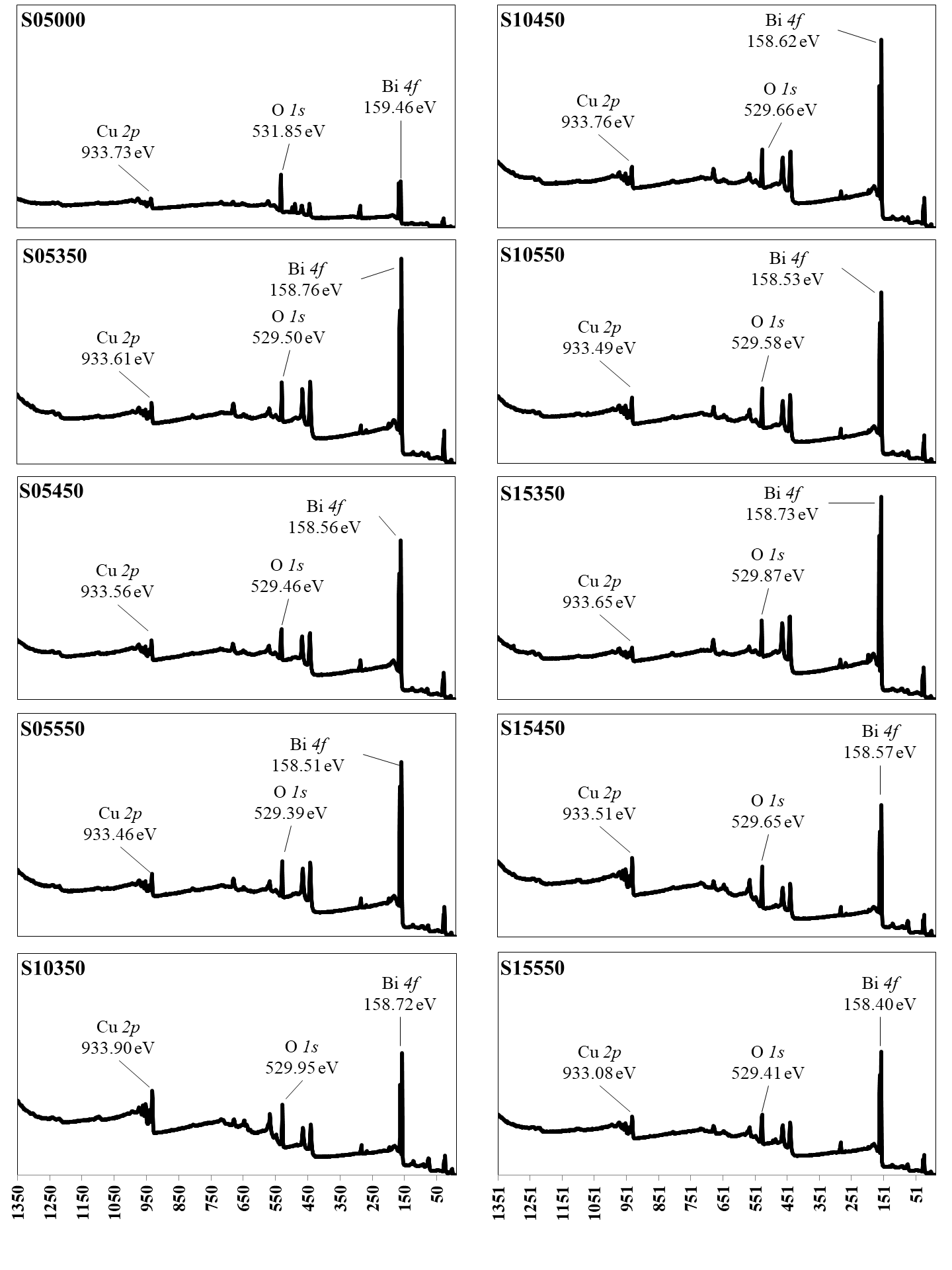


**Figure S6.** The survey XPS spectra of FTO/CuO/CuBi_2_O_4_ photocathodes


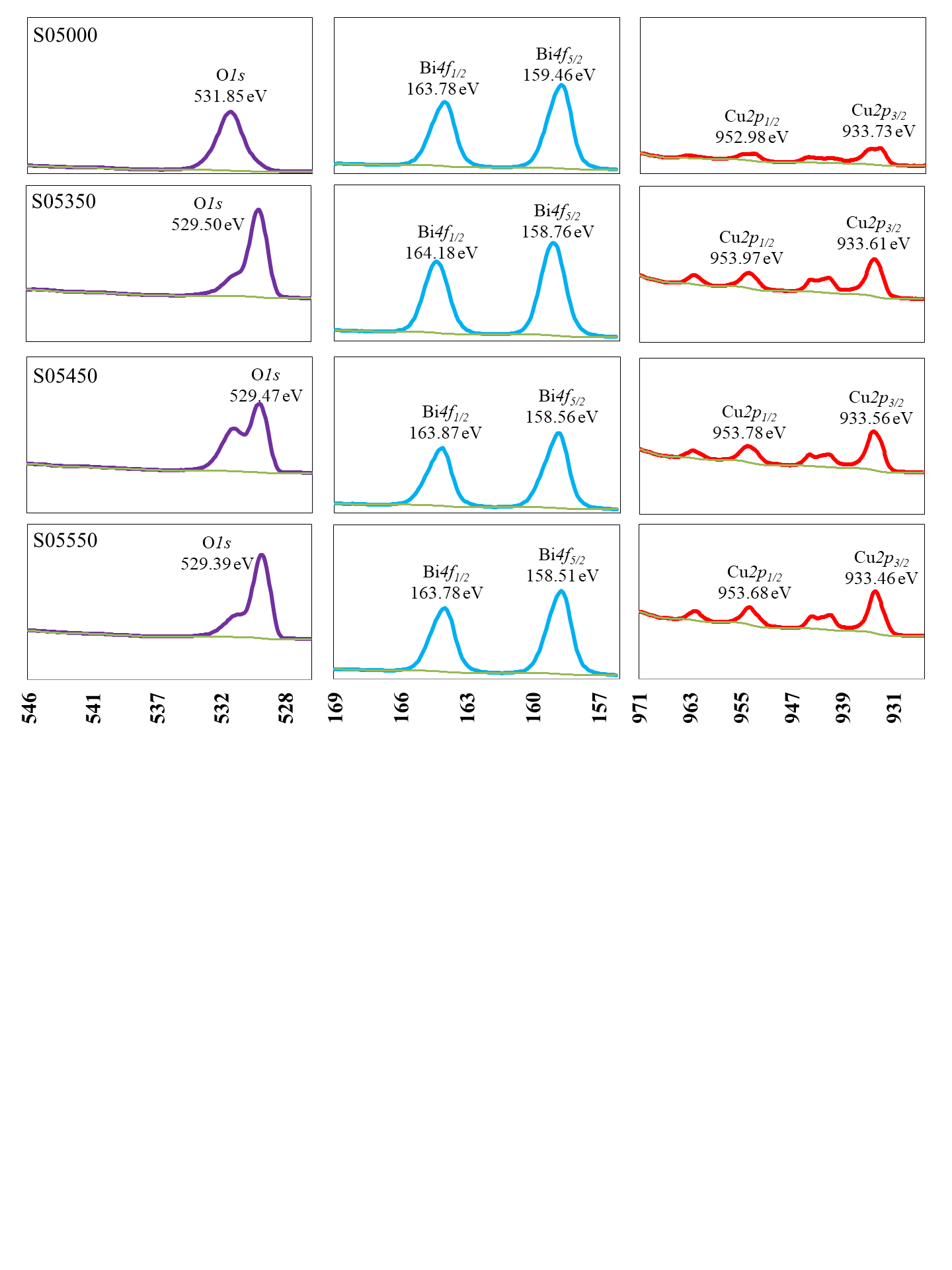

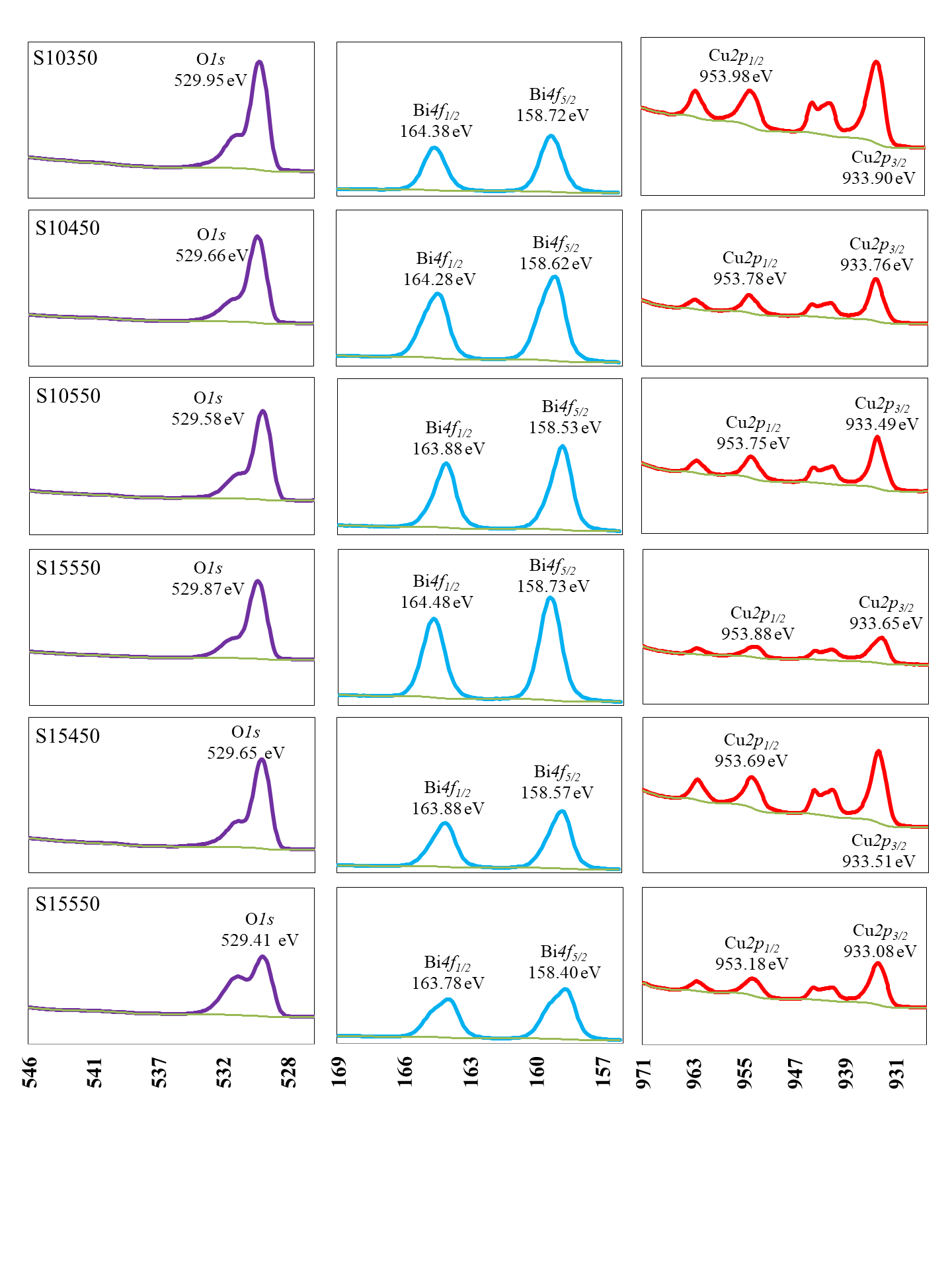


**Figure S7.** High-resolution XPS spectra of FTO/CuO/CuBi_2_O_4_ photocathodes


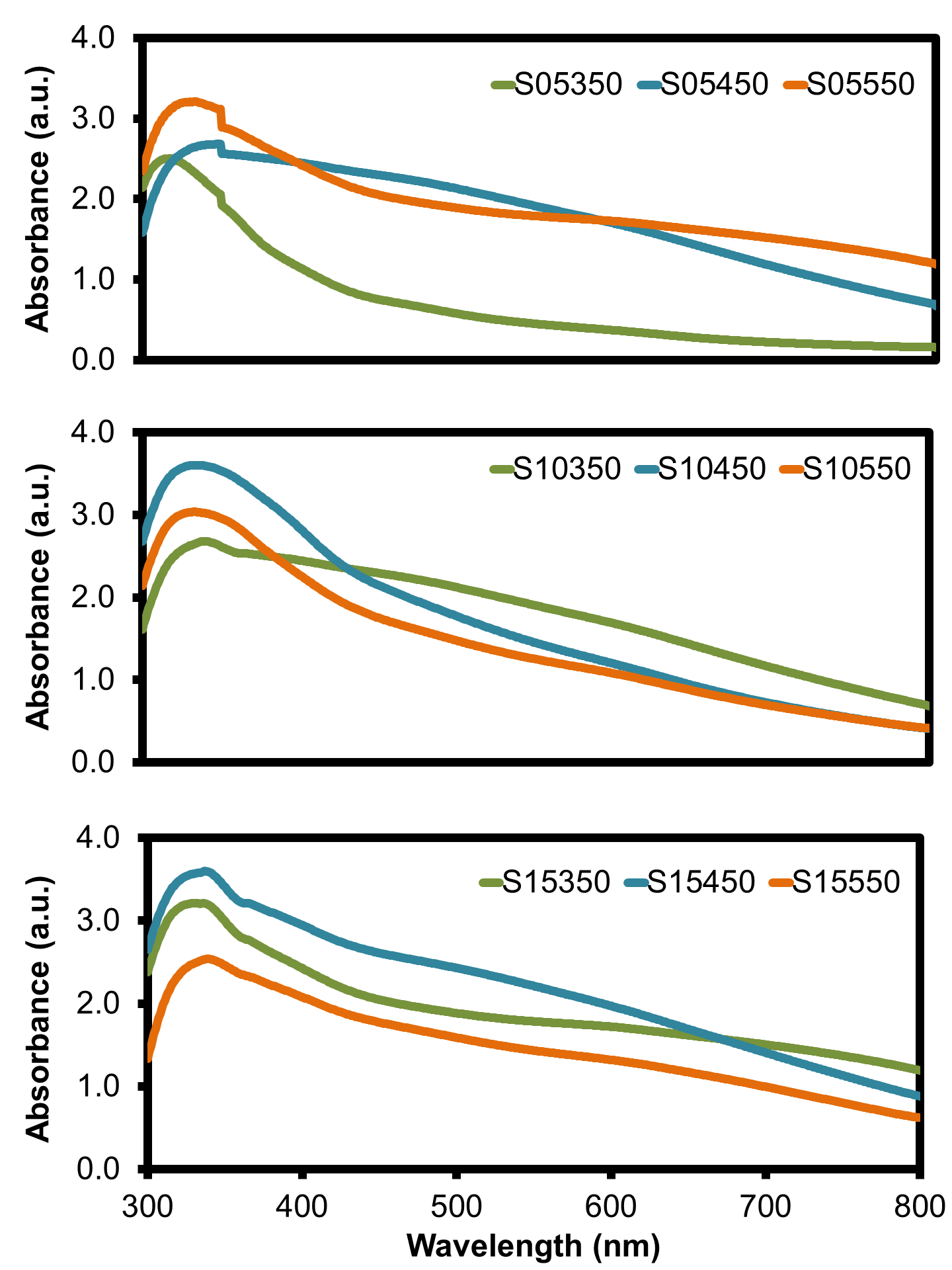


**Figure S8.** UV-Vis absorbance spectra of FTO/CuO/CuBi_2_O_4_ photocathodes


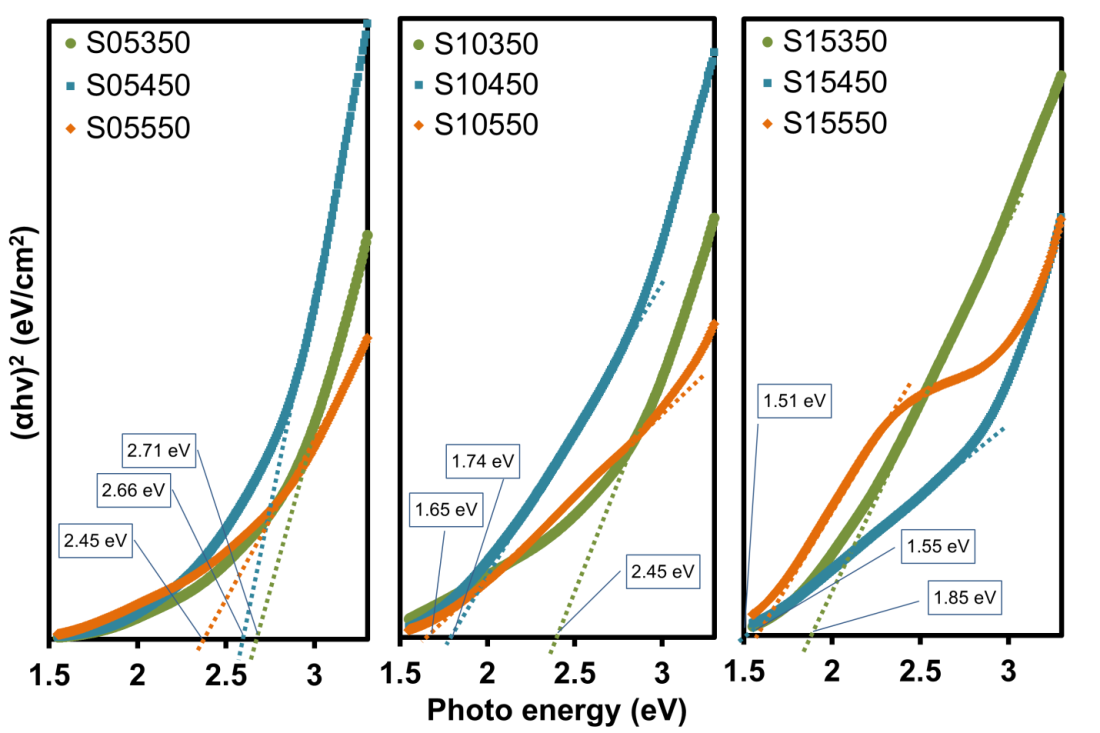


**Figure S9.** Bandgap estimation of FTO/CuO/CuBi_2_O_4_ photocathodes


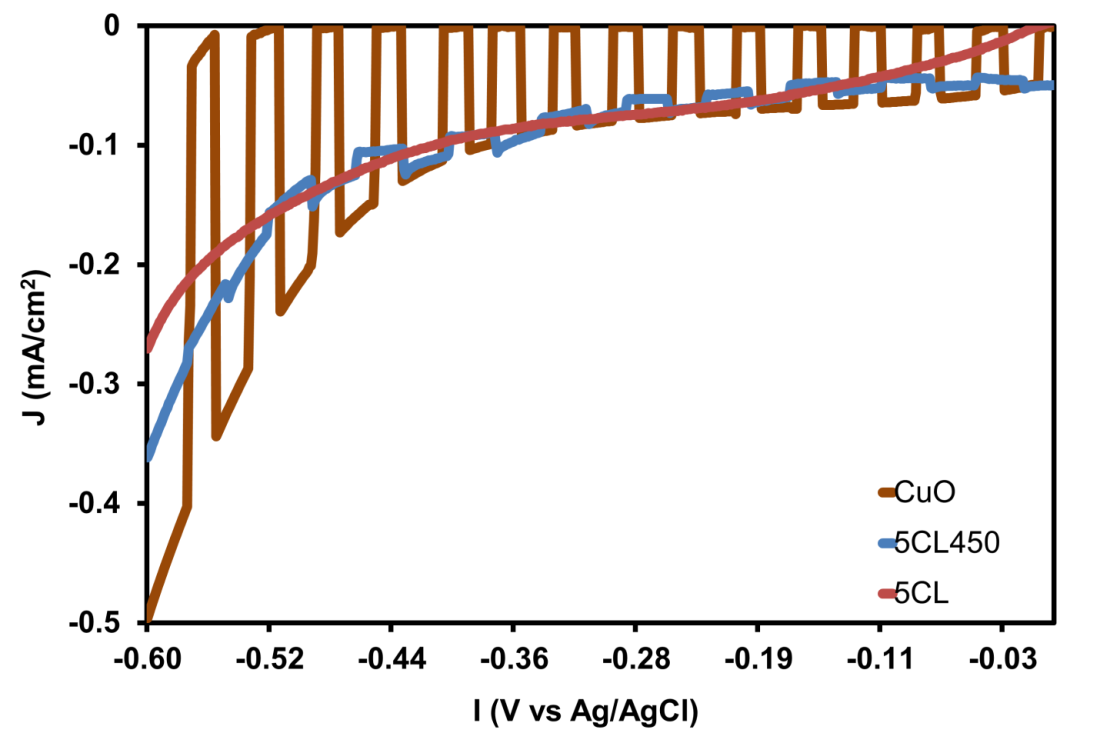


**Figure S10.** The chopped-illumination respond of CuO, 5CL450, and 5CL electrode at -0.6 V potential in 0.1 M Na_2_SO_4_ electrolyte (pH 6.8).


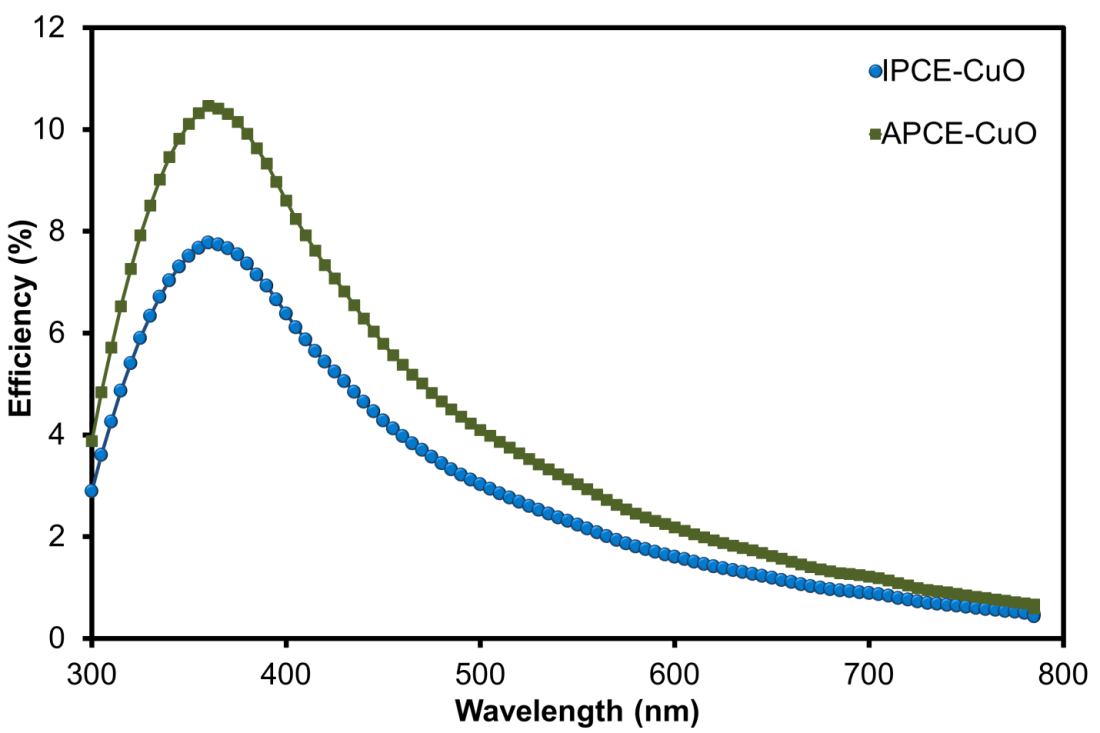


**Figure S11.** The IPCE and APCE of CuO photoelectrode


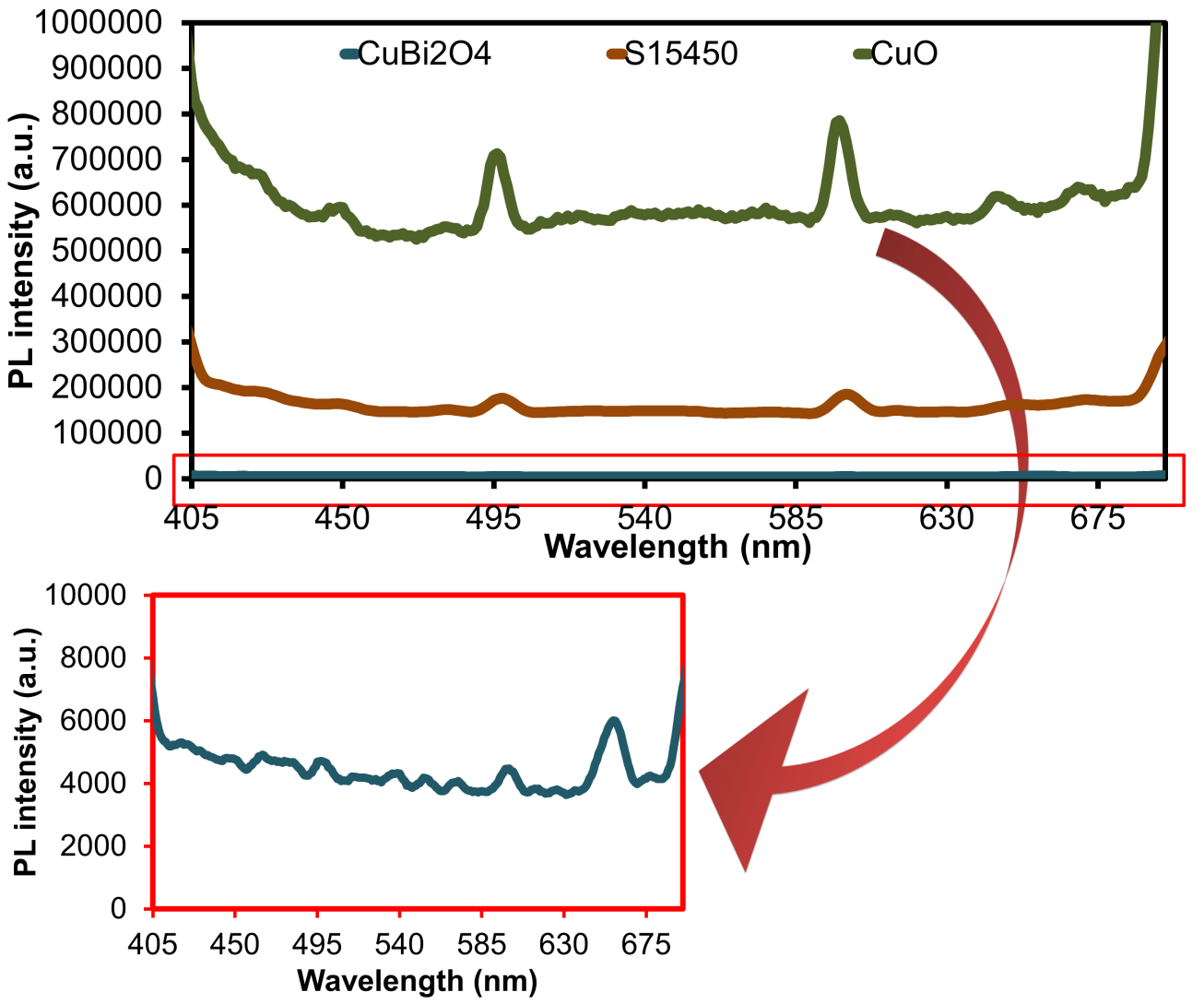


**Figrue S12.** Photoluminescen (PL) spectra of CuO layer, CuBi_2_O_4_ layer, and CuO/CuBi_2_O_4_ photocathode (S15450)
